# Isolation and Characterization of Numerous Novel Phages Targeting Diverse Strains of the Ubiquitous and Opportunistic Pathogen *Achromobacter xylosoxidans*

**DOI:** 10.1371/journal.pone.0086935

**Published:** 2014-01-22

**Authors:** Johannes Wittmann, Brigitte Dreiseikelmann, Christine Rohde, Manfred Rohde, Johannes Sikorski

**Affiliations:** 1 Leibniz Institute DSMZ – German Collection of Microorganisms and Cell Cultures, Department of Microorganisms, Braunschweig, Germany; 2 Department of Microbiology/Genetechnology, Faculty of Biology, University of Bielefeld, Bielefeld, Germany; 3 Helmholtz Centre for Infection Research, Department of Medical Microbiology, Central Facility for Microscopy, Braunschweig, Germany; 4 Leibniz Institute DSMZ – German Collection of Microorganisms and Cell Cultures, Department of Microbial Ecology and Diversity Research, Braunschweig, Germany; Charité-University Medicine Berlin, Germany

## Abstract

The clinical relevance of nosocomially acquired infections caused by multi-resistant *Achromobacter* strains is rapidly increasing. Here, a diverse set of 61 *Achromobacter xylosoxidans* strains was characterized by MultiLocus Sequence Typing and Phenotype MicroArray technology. The strains were further analyzed in regard to their susceptibility to 35 antibiotics and to 34 different and newly isolated bacteriophages from the environment. A large proportion of strains were resistant against numerous antibiotics such as cephalosporines, aminoglycosides and quinolones, whereas piperacillin-tazobactam, ticarcillin, mezlocillin and imipenem were still inhibitory. We also present the first expanded study on bacteriophages of the genus *Achromobacter* that has been so far a blank slate with respect to phage research. The phages were isolated mainly from several waste water treatment plants in Germany. Morphological analysis of all of these phages by electron microscopy revealed a broad diversity with different members of the order *Caudovirales*, including the families *Siphoviridae*, *Myoviridae*, and *Podoviridae*. A broad spectrum of different host ranges could be determined for several phages that lysed up to 24 different and in part highly antibiotic resistant strains. Molecular characterisation by DNA restriction analysis revealed that all phages contain linear double-stranded DNA. Their restriction patterns display distinct differences underlining their broad diversity.

## Introduction

*Achromobacter xylosoxidans* is a nonfermenting, oxidase- and catalase-positive, motile gram-negative rod [1] that can be found widely distributed in natural environments, mainly in moist soil [2] or water sources like well water [3] or swimming pools [4]. It can also be isolated from plants where it shows endophytic and plant growth promoting characteristics [5]–[7]. Additional tolerance of aromatic compounds and heavy metals makes it candidate as an effective bioinoculant for plants in phytoremediation [7] and as a bioremediation tool for contaminated soils [8]–[10]. However, besides some genes for these degradation pathways, its genome reveals also genes associated with pathogenesis, toxin production and antibiotic resistances [10]. Hence, apart from its role as an environmental organism, *A. xylosoxidans* has been recognized during the last years as an emerging nosocomial pathogen. For example, in cystic fibrosis it has become even more prominent than members of the *Burkholderia cepacia* complex [11], although the overall incidence of *Pseudomonas aeruginosa* did not change [12]. It potentially causes a wide range of different human infections also in non-CF patients, including endocarditis [13], [14], bacteremia [15], [16], meningitis [17], ocular infections [18], [19], urinary tract infections [20] and is also an emerging threat for immunocompromised patients [12], [21], [22]. *A. xylosoxidans* is often nosocomially acquired by transmission from patient to patient [23]. Several epidemiological studies also showed that *A. xylosoxidans* is able to survive in uncommon habitats, such as the antisept chlorhexidine [24], or on inanimate surfaces in hospitals [25]. Furthermore, there are reports about outbreaks of infections caused by contaminated dialysis fluids, contrast solutions [26] or ultrasound gels [27], emphasizing its potential as a nosocomially spread opportunistic pathogen. Cases of *A. xylosoxidans* bacteremia with relatively high mortality rates (15–30%) were reported [28], [29] and especially cases of endocarditis caused by catheter related infections with *A. xylosoxidans* show a remarkably high mortality rate (>50%) [13], [30], [31]. In sum, *A. xylosoxidans* has become a serious human pathogen that needs appropriate clinical control. Typically, since the discovery of Penicillin by Alexander Fleming [32] microbial infections have been treated mainly by antibiotics. However, reports on occurring and dramatically developing resistances against antibiotics also in *A. xylosoxidans* [33], [34] mirror the increasing weakness of antibiotics [35], [36] and underline the need to find alternatives to fight against this organism. Several beta-lactamases [34], [37] and resistances against aminoglycosides [38] have been described for *A. xylosoxidans*. Nosocomially acquired bacteria can cause dangerous health threats for individuals or even outbreaks if associated with multidrug-resistances. During the last decade, a revival of bacteriophages as a natural option against bacterial pathogens [39] received new attention [40]–[42]. Bacteriophages are applied in control of bacterial infections in humans, animals and plants. Phages offer some advantages compared to antibiotic therapies: they are highly specific against their target bacteria, self-reproducing at the focus of bacterial accumulation without disturbing the obligate bacterial flora and therefore, without the side effects known for antibiotic drugs. However, until now, phages for *A. xylosoxidans* are hardly known, and the available literature is scarce and old [43], [44]. It is hence unclear if a substantial diversity of phages against *A. xylosoxidans* exists at all and if they potentially could serve as therapeutic phages against this pathogen. Therefore, the leading approach of our investigations was to discover and describe the phage diversity for this species.

In this study, we describe the isolation and characterization of a substantial number and diversity of phages from natural environments that reveal quite a broad host range against a set of in part highly antibiotic resistant *A. xylosoxidans* strains mostly from clinical origin and of different geographical regions throughout Europe.

## Results

### Characterization of *Achromobacter* Strains by MultiLocus Sequence Typing and Phenotype MicroArray

**Figure 1:**
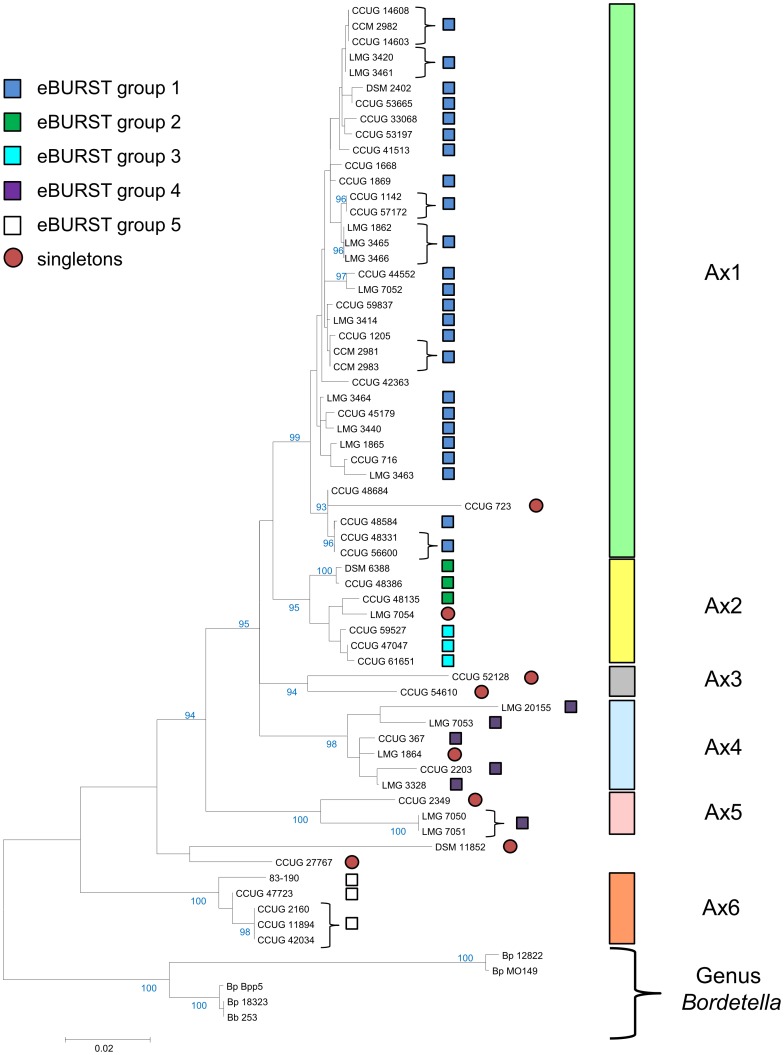
Molecular and phenotypic characterization of the *Achromobacter* isolates. The tree with the highest log likelihood is shown. The percentage of trees in which the associated taxa clustered together, as determined by 500 bootstrap replicates, is shown next to the branches. Only values above 90% are shown. The tree is drawn to scale, with branch lengths measured in the number of substitutions per site. Vertical colored bars indicate robust clades with bootstrap support >90%. Brackets indicate strains that are identical throughout the four partially sequenced genes. Strains that are marked by squared rectangles of the same color belong to the same eBURST group, i.e., share at least one identical locus (gene). Red circles indicate so-called singletons which do not share an identical locus with any of the other strains.

The *Achromobacter xylosoxidans* strains were studied on both a molecular and a phenotypic level in order to study their level of diversity. The sequence analysis of partial sequences of the *rec*A (436 bp, 60 *Achromobacter* strains), *rpo*B (453 bp, 61 strains), *tyr*B (342 bp, 58 strains) and *icd* (430 bp, 61 strains) genes revealed 30, 35, 26, and 35 haplotypes with a mean pairwise sequence divergence (%) of 0.035, 0.022, 0.039, and 0.047, respectively. The concatenation of the four sequences revealed 52 haplotypes. A maximum likelihood phylogenetic analysis revealed six clades Ax1 to Ax6 that were supported by ≥94% bootstrap support. Only two strains, CCUG 27767 and DSM 11852 could not be affiliated to a robust clade. Five eBURST groups were identified, which in general were equivalent to the robust phylogenetic clades (Figure 1). Only very few single locus variants were observed (Supplementary Figure S1), which did not allow to identify any potential founder strain. Clade Ax2 consists of the two eBURST groups 2 and 3. Interestingly, Ax5 strains LMG 7050 and LMG 7051 are members of the eBURST group 4, which predominantly belongs to clade Ax4, indicating that strains LMG 7050 and LMG 7051 share at least one identical gene with some Ax4 strains. Single gene phylogeny indicates that a copy of the *recA* gene of strain LMG 7050 or LMG 7051 (both Ax5) has been horizontally transmitted to the Ax4 strain LMG 7053 (Supplementary Figure S2). In general, however, homologous recombination events do not appear to be frequent among the studied strains, as the null hypothesis of linkage equilibrium among alleles can be rejected clearly (Index of Association I_A_^S^ = 0.121, p<0.001 at 1000 Monte Carlo iterations, suggesting a clonal structure of the *A. xylosoxidans* strains. In general, the molecular based clades identified by maximum likelihood phylogeny are mirrored by the physiological phenotypes as determined by a heatmap analysis using Ward clustering on Euclidian distances of AUC (area under the curve) values from the Phenotype MicroArray analysis (see Supplementary file S1). A principal component analysis suggests that a large number of physiological reactions participate in differentiating the *A. xylosoxidans* strains phenotypically (see Supplementary file S1). Indeed, among 38 out of 96 physiological reactions tested in the Gen III MicroPlates™, a total of 141 significant differences in pairwise comparisons among any of the six Ax groups were observed (see Supplementary file S1).

Overall, both the analysis of the partial sequences of the four genes and the Phenotype MicroArray study suggest a substantial divergence among the studied strains, indicating that we have covered a broad range of diversity within *A. xylosoxidans*.

### Antibiotic Resistance of *Achromobacter*

**Figure 2:**
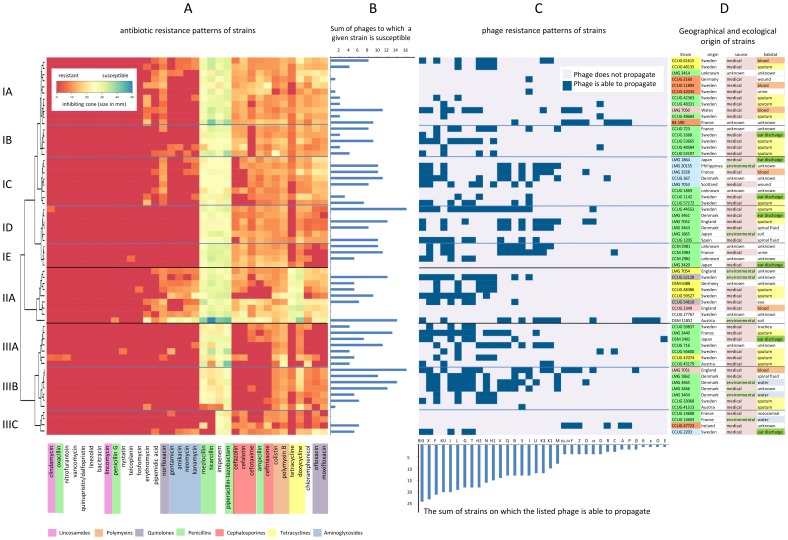
Antibiotic resistance, phage susceptibility and geographic/ecological metadata of *Achromobacter* strains. (A) Quantitative display of susceptibility of 62 strains (rows on the y-axis) to 35 antibiotics (columns on the x-axis) shown as heatmap of antibiotic susceptibility values using Euclidian distance and Ward clustering for both rows and columns as implemented in the heatmap.2() function of the R gplots package [77]. The colour code of the heatmap is given in the upper left corner of the heatmap. The more red the colour, the more resistant is the strain to the antibiotic (for fields covered by the legend, all strains are fully resistant to the respective antibiotic). Only the row dendrogram is shown, which depicts clusters of strains with similar antibiotic resistance patterns (see main text). Antimicrobial agents are grouped according to their clusters of susceptibility patterns of the strains (column dendrogram is not shown). The names of the antibiotics are colored according to their chemical classification. The respective color legend is shown at the bottom of A. (B) Horizontal bar plot which depicts the sum of phages to which a given strain is susceptible (for details see section C). (C) Host range analysis of isolated phages (columns in C). The detailed results of susceptibility of strains per phage are shown as level plot with rows being ordered according to Fig. 2A. A grey color indicates resistance or immunity, blue color indicates plaque formation of a given phage (see the labeling of the x-axis of C) on a given strain (y-axis). The respective legend is shown in the upper right corner of C (for fields covered by the legend, phages do not propagate on the indicated strains). The order of phages (columns of levelplot in C) is based on the number of strains on which a given phage is able to propagate (indicative for the width of the host range). This number of strains is given in the vertical bar plot below the level plot. (D) Names of the *Achromobacter* strains and respective geographic and ecological metadata. Strain names were marked in colors according to clusters from MLSA (see Figure 1), metadata values are differently colored in order to facilitate readability (a colour legend is not separately given).

The susceptibility of the *Achromobacter* strains was tested against 35 different antibiotics by agar diffusion test. In 56% of all antibiotic/strain combinations, a complete resistance with no sign of inhibition at all was observed (Fig. 2A). All strains were completely resistant or at least strongly tolerant against a set of 12 antibiotics, including vancomycin, penicillins such as penicillin G or oxacillin and lincosamides, such as clindamycin and lincomycin (Fig. 2A). The majority of the strains was also resistant to aminoglycosides, e.g. gentamycin or kanamycin. Only four of 35 antibiotics were to some extent inhibitory against the majority of the strains, in particular mezlocillin, ticarcillin, imipenem and piperacillin-tazobactam. The distribution of the resistance spectra was used to classify the strains into three main clusters with several subclusters (I, II and III). The first cluster (I) comprises strains that show the main/typical characteristics of the antibiotic resistance spectra in *Achromobacter* as previously described [33]. It consists of five subclusters (IA-IE) with IA presenting the characteristic resistance spectrum against vancomycin, penicillins and lincosamides mentioned above. The other subclusters can be further characterized by additional resistances against cephalosporine cefotaxime (IB), cephalosporines cefazolin and cefalotin (IC), tetracyclin (ID) and the quinolones ofloxacin and moxifloxacin, chloramphenicol and tetracycline (IE), respectively. The second cluster (II) contains the most sensitive strains of the set examined. These are still susceptible to aminoglycosides, such as neomycin or kanamycin. The third cluster (III) consists of three subclusters and represents the most resistant *Achromobacter* strains: about 70% of the antibiotics were totally effectless in this cluster. Subclusters IIIA and IIIB are highly resistant against all tested cephalosporines, subcluster IIIA strains are additionally resistant to both tetracycline antibiotics. In contrast to subcluster IIIA and IIIB, strains of subcluster IIIC reveal susceptibility towards members of the cephalosporine group, but are additionally resistant to mezlocillin and ticarcillin, which are still inhibitory to basically all other strains. Apparently, neither the geographical origin of strains tested nor the type of habitat (Fig. 2D) correlates with the distribution of antibiotic resistance patterns. Highly resistant strains had been isolated from clinical habitats, such as sputum or blood, as well as from the non-clinical environments. For some phylogenetic clades (Figure 1) there is a good correlation between strain grouping according to phylogeny of gene sequences and clustering according to similarity with respect to antibiotic resistance (Ax2, Ax4, Ax6).

### Broad Host Range and Large Diversity of Novel *Achromobacter* Phages

**Figure 3:**
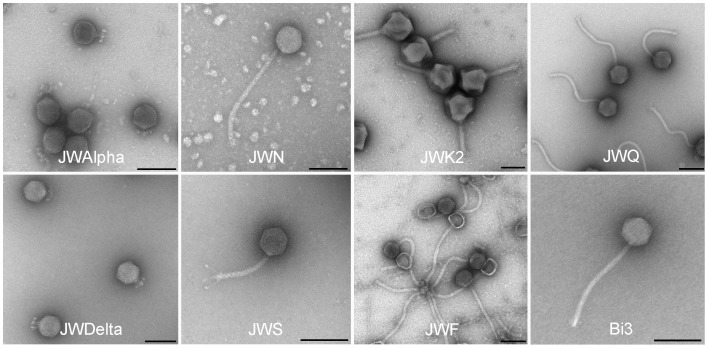
Electron micrographs of several *Achromobacter* phages with different morphotypes. Negative staining (4% (w/v) uranyl acetate, pH 5.0). Bars represent 100 nm.

**Figure 4:**
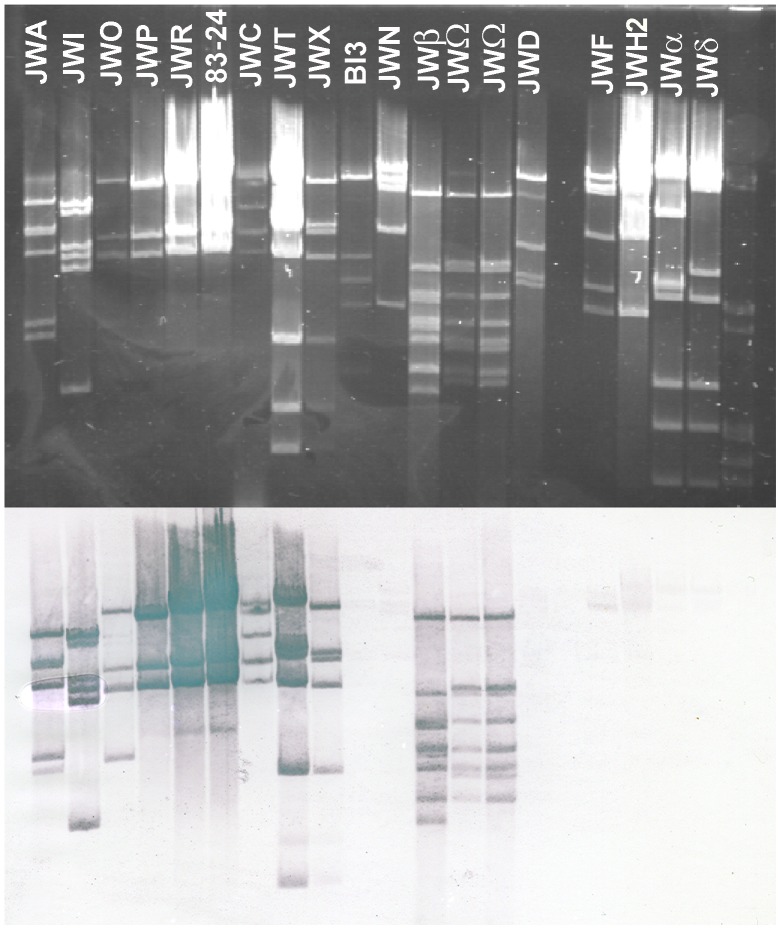
Southern blot hybridization analysis of hydrolyzed genomic DNA of *Achromobacter* phages. Phage DNAs were hydrolyzed with endonuclease NdeI, after agarose gel electrophoresis and Southern blotting, the DNAs were hybridized with DNA from phage 83–24 as a probe. λ DNA hydrolyzed with EcoRI and HindIII was used as marker (M). 1% agarose gel.

Altogether, 34 novel phages were isolated from six samples from three different waste water treatment plants and two soil samples from compost, using 12 different *Achromobacter* strains as hosts (Table 1). The collection of the 61 *Achromobacter* strains proved to be particularly suitable for the host range study of these phages, as they represent a substantial diversity with respect to geographic origin and habitat source. The majority of *Achromobacter* strains (50 out of 61) were lysed by at least one of these phages (Fig. 2B). Per susceptible strain, up to 16 phages were identified to be able to produce progeny phages and lyse cells, with a mean of 5.8 and a median of 5. For all clusters of antibiotic resistance patterns, successfully infecting phages were obtained (Fig. 2B). Per individual phage, up to 24 strains were susceptible, with a mean host range of 10.3 (median 12) host strains per phage, suggesting a quite large host range. Among the 35 phages, 31 different host range patterns were observed, indicating a substantial diversity of phages that use *Achromobacter* as host. This was supported by electron microscopy studies of the morphology of phages (Fig. 3). All examined phages with the exception of phage M belong to the order of *Caudovirales* [45], revealing members of three different families. Most of them display relatively long and flexible tails and therefore belong to the family of *Siphoviridae*. Among this family a variety of different tail lengths and structures was found, most tail lengths ranged from about 130 nm (e.g. JWS) to about 270 nm (e.g. JWF, JWN, JWQ and Bi3) (Table 1). Even the structures of the tail ends varied among these newly isolated phages, revealing short (phage Bi3), long (e.g. phage JWS) or no tail fibers at all (e.g. JWN) (Fig. 3). Also a phage of the *Myoviridae* was isolated, JWK2 shows a large head and a contractile tail, essential properties of this family. Finally, JWAlpha and JWDelta belong to the *Podoviridae* and have only short and stubby tails. This high diversity in host range and morphology was further supported by molecular characterization through restriction endonuclease digest of their double-stranded DNA. This is especially noteworthy for phages of identical host range or morphology. For instance, phages JWA and JWP both lyse the same two host strains (CCUG 47723 and 83–190; Fig. 1C) and have also a very similar morphology as members of the *Siphoviridae* (Fig. 3), their DNA restriction patterns identify them as two different phages (Fig. 4). Hybridization of phage 83–24 DNA as a probe against different phage DNAs of our set revealed a probably strong relatedness of different phages on nucleotide level (Fig. 4.), distinct signals over the whole range of restriction fragments could be detected in 10 of 17 cases (∼58,8%). All signals referred to phages of the Siphoviridae, no signals were detected in lanes with DNA of podoviruses.

**Table 1:** Overview and metadata of isolated environmental phages.

Phage	Origin	Hoststrain	Monthsampling	Morphotype	Putativephage life-cycle	Plaquemorphology	Reinduction	Headlength	Taillength
JWα	W	DSM 11852	05/2012	Podovirus	virulent	clear	No	59 nm	22 nm
JWβ	B	DSM 11852	06/2012	Siphovirus	virulent	clear	No	60 nm	121 nm
Bi3	Bi	DSM 11852	09/2012	Siphovirus	temperate	turbid	Yes	62 nm	241 nm
JWδ	B	DSM 11852	05/2012	Podovirus	virulent	clear	No	72 nm	22 nm
JWε	B	DSM 11852	07/2012	Siphovirus	virulent	clear	No	60 nm	230 nm
JWΩ	B	DSM 11852	08/2012	Siphovirus	virulent	clear	No	60 nm	124 nm
JWA	B	83–190	08/2012	Siphovirus	virulent	clear, small halo	No	59 nm	127 nm
JWB	B	CCM 2983	07/2012	Siphovirus	virulent	clear	No	60 nm	120 nm
JWC	B	83–190	07/2012	Siphovirus	virulent	clear, small halo	No	62 nm	138 nm
JWD	B	CCUG 48331	07/2012	Siphovirus	virulent	clear	No	70 nm	265 nm
JWE	B	DSM 2402	05/2012	Siphovirus	temperate	turbid	Yes	60 nm	120 nm
JWF	B	CCUG 48386	06/2012	Siphovirus	temperate	turbid	Yes	62 nm	254 nm
JWG	B	CCUG 52128	07/2012	Siphovirus	temperate	turbid	Yes	59 nm	124 nm
JWH1	B	CCUG 47074	07/2012	Siphovirus	temperate	turbid	Yes	67 nm	306 nm
JWH2	B	CCUG 47074	07/2012	Siphovirus	temperate	Clear, with manydistinct microcolonies	Yes	78 nm	303 nm
JWI	B	CCUG 41513	06/2012	Siphovirus	virulent	clear, halo	No	60 nm	140 nm
JWJ	B	CCUG 52128	07/2012	Siphovirus	temperate	turbid	Yes	59 nm	162 nm
JWK1	B	LMG 3328	07/2012	Siphovirus	temperate	turbid	Yes	78 nm	262 nm
JWK2	B	LMG 3328	07/2012	Myovirus	temperate	turbid	Yes	135 nm	142 nm
JWKU	B	LMG 3328	07/2012	Siphovirus	temperate	turbid	Yes	71 nm	262 nm
JWL	B	CCUG 48386	07/2012	Siphovirus	temperate	turbid	Yes	70 nm	270 nm
JWM	B	LMG 20155	07/2012	NA	virulent	Diffuse, after 2 days	No	71 nm	Not detectable
JWN	B	CCUG 48135	07/2012	Siphovirus	temperate	turbid	Yes	74 nm	288 nm
JWO	B	83–190	06/2012	Siphovirus	virulent	clear, small halo	No	62 nm	138 nm
JWP	B	83–190	08/2012	Siphovirus	virulent	clear, small halo	No	57 nm	140 nm
JWQ	B	LMG 3465	08/2012	Siphovirus	temperate	turbid	Yes	78 nm	270 nm
JWR	B	83–190	06/2012	Siphovirus	virulent	clear, small halo	No	63 nm	140 nm
JWS	B	CCM 2983	06/2012	Siphovirus	temperate	turbid	Yes	59 nm	138 nm
JWT	B	LMG 3465	08/2012	Siphovirus	virulent	clear	No	61 nm	133 nm
JWU	B	LMG 7052	07/2012	Siphovirus	temperate	turbid	Yes	76 nm	276 nm
JWV	B	LMG 7052	07/2012	Siphovirus	temperate	turbid	Yes	70 nm	250 nm
83–24	L	83–190	–	Siphovirus	virulent	clear	No	60 nm	140 nm
JWX	B	LMG 3465	08/2012	Siphovirus	temperate	turbid	Yes	62 nm	128 nm
JWY	B	83–190	05/2012	Siphovirus	virulent	clear	No	75 nm	161 nm
JWZ	B	83–190	05/2012	Siphovirus	virulent	clear	No	58 nm	130 nm

The two podoviruses JWAlpha and JWDelta which show a very similar N4-like morphology, display similar though not identical DNA restriction fingerprints (Fig. 4, also supported by other restriction digests not shown), suggesting them to be closely related. Interestingly, these phages were isolated from two different waste water treatment plants with quite a geographical distance (about 250 km between Braunschweig and Werl), suggesting a more global distribution of this phage type. Also, with respect to clusters of antibiotic resistance patterns, the host range among the isolated phages was broad. There is no preference of a given phage for host strains of a specific antibiotic resistance cluster. Some of the phages (e.g. phage Bi3 or KU) were able to lyse strains from different *Achromobacter* species (Fig. 2C). Altogether, in combining studies of morphology, host range and DNA restriction patterns, we could show that this set of newly isolated phages contains 34 different phages with a large diversity in all studied aspects. Similarly to the antibiotic resistance patterns, also no correlation between phage susceptibility and either geographical origin or habitat of isolation could be observed (Fig. 2D).

### Frequency and Characteristics of Surviving Cells after Phage Treatment

Some newly isolated bacteriophages were able to form plaques on a relatively wide range of strains. In order to examine their lysis behaviour and efficiency, we performed an exemplary determination of the frequency of spontaneously emerging bacteriophage-insensitive mutants (BIMs) or lysogens with three relatively broad host range phages (Bi3, JWF and JWX) using bacterial strains on which all phages were able to form plaques. This study revealed that despite a high multiplicity of infection (>100) there was still a high number of resistant or lysogenic cells after treatment with these bacteriophages.

Same experiments with phage cocktails using combinations of two or all three phages Bi3, JWF and JWX resulted in a decreased frequency of BIMs (Table 2). Further examination of putative mutants in respect to a possible resistance against or lysogenization by phages applied in this experiment showed that the applied phages might be temperate and follow the lysogenic life-cycle (Table 1), as treatment of BIMs with UV irradiation resulted in plaque formation in some cases. This assumption was further supported by the turbid plaque morphology of these phages. Despite an already existing prophage, e.g. phage JWF seemed to be able to integrate into the genome of strain CCUG 48386 that was originally used for phage propagation. Treatment with UV light resulted in induction of plaque forming of phage JWF. Phage JWX could also be induced again from a BIM of strain CCUG 367 that had revealed no sign of a prophage in induction experiments before. Integration of temperate phages into the genome of the host cell does not seem to be quite stable as phages could also be detected in the supernatant of uninduced cultures of BIMs. In general, most mutants that were tested for phage susceptibility after the first treatment with phages, were slowly growing, but still susceptible to phages. Plaque morphology and/or re-induction experiments of all isolated phages led to the assumption that half of them seem to be temperate (Table 1). With the exception of phage JWH2, all reinducible phages revealed turbid plaques. Only full genome sequencing would allow to draw more secure conclusions on the life style, e.g. by identifying putative integrases or repressors.

**Table 2:** Determination of the frequency of bacteriophage-insensitive mutants or lysogens after phage treatment.

strain	phages						
	Bi3	JWF	JWX	Bi3/JWX	JWF/JWX	Bi3/JWF	Bi3/JWF/JWX
CCUG52128	0.391	0.0328	0	0	3.9×10^−4^	0.74	0
CCUG367	1	0.0231	1	1.1×10^−3^	6.6×10^−4^	0.161	0.02
CCUG59527	1	1	0	0.0261	0	0.1164	4.76×10^−4^

Putative life cycles of the isolated phages were assumed after determination of plaque morphology and reinduction experiments.

Mean numbers of viable counts in one ml of culture after phage treatment were divided by mean numbers viable counts without phage treatment. “0” describes the total elimination of all cells, “1” stands for no significant elimination.

## Discussion

The purpose of this study was to isolate and characterize novel phages targeting *Achromobacter xylosoxidans*. Apart from its role as an environmental organism, during the last years, *A. xylosoxidans* has been increasingly recognized as nosocomial pathogen [11]–[18], [21], [22], in particular for immunocompromised patients [29]. Knowledge on *Achromobacter* phages is scarce and from publications more than 30 years old [43], [44], motivating us to perform a comprehensive search for *Achromobacter* phages. In order to characterize the host-range of novel phages, we investigated a set of 61 *A. xylosoxidans* strains that were shown by molecular and phenotypic characterization to represent a broad diversity (Fig. 1, Supplementary file S1). As the identification of potential therapeutic phages targeting multiple antibiotic resistant pathogens is becoming increasingly an issue in medicine, biotechnology or food production [46]–[50], we additionally investigated the distribution of antibiotic resistances in these strains.

The antibiotic resistance spectra of 61 *A. xylosoxidans* strains from different environmental and clinical sources showed similar multi-resistance profiles as compared to other strains of this species described previously. To some extent, phylogenetically closely related strains displayed similar antibiotic resistance characteristics, such as clades Ax4, Ax6, and, in part, clade Ax2. The dominant clade Ax1 is characterized by a large variety of different antibiotic resistance patterns. Interestingly, in our study, about 80% of all tested strains were totally resistant against amikacin, whereas Reddy et al. [19] still report a sensitivity of *Achromobacter xylosoxidans* against amikacin, suggesting this antibiotic, besides some others, to be suitable for treatment of keratitis. The difference in these results may be explained by an ecologically more narrow set of strains investigated in the Reddy et al. [19] study, mainly from a single hospital, as compared to our set of strains. The most active antibiotics in our study were piperacillin-tazobactam, ticarcillin, mezlocillin and imipenem, representing broad spectrum penicillins and a carbapenem, respectively. Looking at the sources or habitats the strains were isolated from, there seems to be no correlating pattern between these factors and the number of antibiotic resistances the examined strains revealed. Besides some innate antibiotic resistances, Traglia et al. [51] report a variety of genetic elements in *A. xylosoxidans* strains, e.g. IncP plasmids and integrons, that probably contribute to the distribution of resistance genes and make *A. xylosoxidans* a reservoir for spreading antibiotic resistances through horizontal gene transfer. Also, the genome sequence of strain A8 reveals several phage-related genes [10].

Phages play a role in horizontal gene transfer by general transduction or integration by recombination as prophages into the host genome. Their probable role as gene vehicles could also support the exchange of genes responsible for antibiotic resistances between different strains and even species [52]. An unstable generalized transduction of auxotrophic markers by phages in *Achromobacter* was reported by Woods and Thomson [53]. Though there is currently no further information available on the prophages identified in this study, it cannot be excluded that prophages may serve as vehicles for antibiotic resistance in *Achromobacter*. As *Achromobacter* appears to be environmentally wide spread [2], [3] we assumed waste water treatment plants to be an appropriate habitat to look at. This habitat is a rich source for bacteriophages against a vast range of bacterial genera [54]–[56]. In this study, the isolation of over 30 different and so far unknown phages from 8 different samples from waste water treatment plants in three different German cities, Braunschweig, Bielefeld and Werl and from soil showed that the pool of phages for this genus appears to be large and to be isolated rather easily; each sample contained at least three different phages when more than two different strains were used for isolation. There is no MLST clade (Fig. 1) or antibiotic resistance cluster (Fig. 2) that appears particularly sensitive or resistant to any of the newly isolated phages, indicating a fairly rapid evolution of phage sensitivity or resistance of *A. xylosoxidans* strains. Still, there are individual strains that appear rather sensitive or resistant to a broader range of phages (Fig. 2). Identifying members of several families of the order *Caudovirales* in our set of newly isolated *Achromobacter* phages correlates with the most recent detailed statistics on the distribution of phage morphotypes in different bacterial genera [57]. Many Gram-negatives (*Enterobacteriaceae*, *Aeromonas*, *Pseudomonas*) have a balanced phage population, comprising *Myo*-, *Sipho*-, and *Podoviridae* as well as phages with cubic and filamentous symmetry. Our study is the first revealing such a large number and diversity of phages in regard to host range, morphology, and genetic diversity in the species *Achromobacter xylosoxidans*. Three phages may further exemplify this. First, the virulent phages JWAlpha and JWDelta share a similar morphology as members of the *Podoviridae* and first amino acid sequence analyses classify them as N4-like phages (unpublished). *E. coli* phage N4 was a genetic orphan for more than 40 years, being provided with a virion-encapsulated RNA polymerase of its own for transcription of its early genes [58]. In recent years, more phages related to N4, but with genetic differences were isolated against other species [59]. Revealing certain similarities in morphology and in particular, in their genomic structure by their DNA restriction fingerprint patterns despite some geographical distance, these two phages seem to have distinct evolutionary origins and might also give more insight into the N4 family. Second, phage M might be an interesting candidate for further studies on different aspects of phage research. Morphological analysis did not reveal any tail-like structure, which makes it a possible member of untailed phage families. Moreover, it is the only phage of this set that needs more than 24 h for plaque formation which lets us assume that it probably has developed an adapted mechanism of host lysis. Furthermore, the siphoviruses of our study also reveal aspects calling for further examination. Though phage 83–24 is the only phage of our study that was not isolated from our samples and therefore might not be closely related to the others, Southern hybridization of its DNA against other phage DNAs of our investigations showed that it is strongly related to some other phages of this study, including temperate and virulent ones. Most phage genomes are organized in modules that can be exchanged among a phage population by recombination. These phages might also be interesting examples of this so-called mosaicism in bacteriophage genomes [60]. Further examined phages JWF, JWX and Bi3 showed a relatively broad host range, but also reveal characteristics of temperate phages as they seem to be able to integrate into the host genome which was shown be re-induction tests via UV irradiation. Tests for control of different *A. xylosoxidans* strains with these phages showed that apart from phage JWX, their lysis rates were not highly efficient when only one phage was applied. Cocktails of two or three phages enhanced efficacy in most cases, but complete eradication of bacterial cells was achieved only in few cases. Because of a decreased efficacy of phage cocktails in comparison with single phage approaches in some cases we assume that these phages appear to be temperate and in some way might prevent a successful eradication by other phages after integration into the host genome. Interestingly, phages JWX and JWT show nearly identical DNA restriction patterns, whereas plaque morphology and reinduction experiments suggest them to be temperate and virulent, respectively. Since they were isolated from the same sample, phage JWT might be a mutated version of JWX containing mutations in the genes for an integrase or a repressor that make it unable to integrate and remain in the host genome. This finding makes these phages interesting candidates for sequence analysis and comparison in the future.

## Conclusions

This study shows for the first time that a large diversity of phages targeting a broad range of *A. xylosoxidans* strains is existing in the environment and can also readily be isolated. The propagation of several of the phages on numerous antibiotic resistant strains of *Achromobacter* nourishes the hope to find potential candidates for therapeutic application to bacterial infections caused by *Achromobacter.* However, it is still unclear if any of the here described phages are suitable therapy phages. Further steps to investigate this would include full genome sequencing in order to exclude the presence of genes for toxins and to verify the absence of genes that might play a role in lysogeny. Additionally, testing of suitable virulent phages under conditions reflecting better a medical environment, such as artificial sputum medium [61] or infected lungs of mice [62] might support and encourage investigations towards possible future applications.

## Materials and Methods

### Ethics Statement

Soil samples were taken on private land with permission of the owner. Water samples were taken from waste water treatment plants, no specific permissions were needed as these were public, but we announced them and informed field workers on-site, for sure. We confirm that field studies did not involve endangered or protected species.

### Bacterial Strains and Growth

The 61 *Achromobacter* strains examined in this study were obtained from five culture collections internationally renowned (Culture Collection, University of Göteborg (CCUG), Sweden; Czech Collection of Microorganisms (CCM), Czech Republic; Felix d’Herelle Reference Center for Bacterial Viruses, Université Laval, Canada; Leibniz Institute DSMZ, Germany and LMG Bacteria Collection, Belgium). The strains were isolated mainly in Europe and represent a wide set of predominantly clinical habitats like sputum, blood, urine or ear discharge, most of the environmental strains had been isolated from soil or water (Supplementary Table S1). In respect to their geographical origin, the majority of the strains (∼41%) was isolated in Sweden, the others originated from different countries in Western Europe besides some strains from Japan and the Philippines. The Swedish strains with clinical origin varied substantially in regard to the year of isolation, clinical habitat or age and sex of patients. Following the information given by the culture collections, the strains were classified predominantly to *A. xylosoxidans* (N = 55), three strains to *Achromobacter ruhlandii*, two strains to *Achromobacter denitrificans* and one unclassified *Achromobacter sp*. strain. However, in a maximum likelihood analysis of partial sequences of four housekeeping genes the non *A. xylosoxidans* strains do not form separate clusters but mingle inseparably with *A. xylosoxidans* strains (this study). We therefore regard all strains analyzed in this study to be members of *A. xylosoxidans*, however, without attempting any taxonomic classification and valid nomenclature. Unless reported differently, all strains were cultured in liquid tryptic soy broth TSB (30 g^−l^, pH 7.5, OXOID) medium or on petri dishes of TSB medium supplemented with 1.5% agar (w/v) for approximately 16 h (overnight) at 28°C.

### Molecular Characterization of *A. xylosoxidans* Strains

The evolutionary history was inferred by using the Maximum Likelihood method based on the Jukes-Cantor model [63]. Initial tree(s) for the heuristic search were obtained by applying the Neighbor-Joining method to a matrix of pairwise distances estimated using the Maximum Composite Likelihood (MCL) approach. A discrete Gamma distribution was used to model evolutionary rate differences among sites (5 categories (+*G*, parameter = 0.6316)). The rate variation model allowed for some sites to be evolutionarily invariable ([+*I*], 41.6056% sites). The tree is drawn to scale, with branch lengths measured in the number of substitutions per site. The analysis involved 66 nucleotide sequences. All positions with less than 0% site coverage were eliminated. That is, fewer than 100% alignment gaps, missing data, and ambiguous bases were allowed at any position. There were a total of 1661 positions in the final dataset. Evolutionary analyses were conducted in MEGA5 [[64]]. The haplotype assignment and estimation of sequence diversity of individual genes was performed using DNaSP version 5.10 [65]. The sequence type pattern was analysed using eBURST [66], with a minimum of one identical locus chosen for the threshold defining an eBURST group definition. The Index of Association I_A_^S^ among alleles was determined using LIAN 3.0 [67]. *Bordetella parapertussis* strains Bpp5 (NC_018828.1) and 12822 (NC_002928.3), *Bordetella pertussis* 18323 (NC_018518.1) and *Bordetella bronchiseptica* strains 253 (NC_019382.1) and MO149 (NC_018829.1) were chosen as an outgroup.

### Phenotypic Analysis using the Phenotypic MicroArray Technology

Bacterial strains for the microtiter plate assay were cultured as described. All strains were examined phenotypically by using a tetrazolium dye oxidation-reduction growth system developed by Biolog, Inc. Single colonies were picked and suspended in inoculation medium IF-A (Biolog) and then inoculated into Gen III MicroPlates™ according to the recommendations of the manufacturer. The plates were incubated in the OmniLog™ incubator at 28°C for 96 h. Every 15 minutes the change of the redox dye values was automatically measured and recorded in order to determine the respiration curve kinetics. For each strain, two independent biological replicates were performed. The data were analysed using the R package opm [68], [69]. From the obtained curve kinetics, the area under the curve (AUC) was determined using the spline-fit algorithms as implemented in the opm function *do_aggr()*. The AUC values were analysed by a heat map clustering using the opm *heat_map()* function under default conditions (euclidian distance, Ward clustering). The AUC values were further analysed by a principal component analysis using the *rda()*, *biplot()*, *ordisymbol()*, and *ordiequilibriumcircle()* functions from the R packages vegan and BiodiversityR [70], [71]. In order to identify significant differences between any of the clades as identified by maximum likelihood phylogeny at the level of individual wells, the opm function *opm_mcp()* was used, which automatically corrects for the risk of type I errors when comparing multiple groups [72], [73].

### Amplification and Sequencing

PCR amplification of partial regions for four housekeeping genes (*rec*A, *tyr*B, *rpo*B and *icd*) and subsequent sequencing were carried out with primers as described by Ridderberg et al. [23] with minor modifications. Cycling parameters were modified from Ridderberg et al. [23] to the following: 95°C for 3 min, followed by 29 cycles of the appropriate annealing temperature (55°C for *rpo*B and *rec*A, 58°C for *icd* and *tyr*B) for 1 min, 2 min at 72°C, followed by 1 min at 95°C with a final extension for 5 min at 72°C. Sequencing was performed with PCR forward primers on an ABI 3500 Genetic Analyzer (Applied Biosystems®) using the Sanger method. All sequence chromatograms were manually checked for accuracy to exclude mistakes and ensure high sequence quality. No PCR amplicons could be obtained from strain CCUG 48684 for the *rec*A gene and from strains CCUG 42363, CCUG 1668, and CCUG 47047 for the *tyr*B gene.

### Nucleotide Sequence Accession Numbers

DNA sequences were deposited in Genbank under accession numbers KF678868-KF678928 (*rpo*B), KF678929-KF678989 (*icd*), KF678990-KF679047 (*tyr*B) and KF679048-KF679107 (*rec*A).

### Antibiogram

The susceptibility of the *Achromobacter* strains towards antimicrobial substances (OXOID) was examined by agar disc diffusion assay according to German standard DIN 58940 which agrees with EUCAST. In order to obtain plates with a homogeneous bacterial lawn, 1 ml of a diluted (10^−3^) overnight culture was spilled on one plate for inoculation and the supernatant was then directly removed. After drying the inoculated plate in a microbial safety cabinet class II, antibiotic discs were placed on agar plates. The antibiotic discs included penicillin G (10 IE), oxacillin (5 µg), ampicillin (10 µg), ticarcillin (75 µg), mezlocillin (30 µg), cefalotin (30 µg), cefazolin (30 µg), cefotaxime (30 µg), imipenem (10 µg), tetracycline (30 µg), chloramphenicol (30 µg), gentamicin (10 µg), amikacin (30 µg), vancomycin (30 µg), erythromycin (15 µg), lincomycin (15 µg), ofloxacin (5 µg), norfloxacin (10 µg), colistin (10 µg), pipemidic acid (20 µg), nitrofurantoin (100 µg), bacitracin (10 IE), polymyxin B (300 IE), kanamycin (30 µg), neomycin (30 µg), doxycycline (30 µg), ceftriaxone (30 µg), clindamycin (10 µg), fosfomycin (50 µg), moxifloxacin (5 µg), linezolid (30 µg), nystatin (100 IE), teicoplanin (30 µg) and the compounds quinupristin/dalfopristin (15 µg) and piperacillin-tazobactam (40 µg). After incubation at 28°C for 16 h the diameter of the inhibition zones was measured.

### Induction of Prophages

Single colonies of putative lysogenized strains after phage treatment were streaked on Petri dishes of TSB medium supplemented with 1.5% agar (w/v) and incubated for approximately 6 h. After that they were irradiated with UV light (UV hand lamp, Köhler, Neulußheim, Germany, order number E-1450) at 254 nm for 20 s from 15 cm distance (∼500 µW/cm^3^). After irradiation, agar plates were overlayed with soft agar (0.5% agar (w/v) containing log phase cells of the original, non-lysogenized bacterial strain. After incubating at 30°C overnight (approximately 16 hours), lawns were checked for plaques.

### Isolation of Bacteriophages

Several waste water samples from three waste water treatment plants (taken in 2012 on: May 8^th^, June 15^th^, July 26^th^, August 21^st^, all Braunschweig, September 20^th^, Bielefeld, May 15^th^, Werl) were used to isolate new phages by utilizing 12 *Achromobacter* strains of this study as hosts. Two soil samples (20 g) from compost from a field were suspended in 40 ml of buffer (10 mM Tris HCl, pH 7.5, 50 mM NaCl, 1 mM CaCl_2_, 1 mM MgCl_2_) each. After gentle shaking for 2 h, the samples were centrifuged to remove sedimented particles and then filtered (membrane syringe filter 0.45 µm, Sartorius, order number 16555). The water samples were filtered directly. For enrichment of bacteriophages, 1 ml of culture of the indicator strains, grown overnight for approximately 16 h, was mixed with 30 ml of liquid TSB (30 g^−l^, pH 7.5, Oxoid) including 1 mM CaCl_2_ and MgCl_2_ and 10 ml of filtered sample. After incubation overnight (16 h) at approximately 20°C, bacteria were removed by centrifugation (20 min, 15.000 g, 50 ml tubes), the supernatant was filtered (membrane syringe filter 0.45 µm, Sartorius, order number 16555) and aliquots were dropped on TSB plates with bacterial lawns of the same strain. Single plaques were further purified by dilution steps and repeated single plaque isolation (see above). Phages were then propagated on their host strains. Phage 83–24 was obtained from the phage collection of Felix d’Herelle Reference Center for Bacterial Viruses at Laval University (Table 1).

### Host Range Analysis of Phages

The host range of all isolated *Achromobacter* bacteriophages was tested on all 61 *Achromobacter* strains. Phages lysates were diluted in liquid TSB medium and 10 µl aliquots were spotted on indicator plates (TSB) overlayed with 3 ml TSB soft agar (0.75% agar) containing 100 µl log phase cells of *Achromobacter*. Plates were incubated at 30°C for 24 h. To ensure that lysis zones are not the result of bacteriocins, lysis of strains was defined as appearance of single plaques upon appropriate dilution of the phages.

### Determination of Bacteriophage-insensitive Mutant Frequency

The frequency of emergence of bacteriophage-insensitive mutants (BIMs) was determined with standard protocols [74]. Bacterial cells were incubated with phages with a multiplicity of infection (moi) of 100 for 45 min to allow proper adsorption of the phages. The mixtures were then diluted, plated on agar plates and incubated at 28°C for 48 h. All experiments were performed in duplicate.

### Purification of Phages and Phage DNA

Purification of the phages by CsCl gradient centrifugation and of their DNA by phenol extraction was done as previously described [75].

### Southern Blot and Hybridization

Southern blotting of hydrolysed phage genomes was performed with a vacuum blotter (Pharmacia, VacuGene XL) on a nylon membrane (Macherey & Nagel, porablot NY amp). Labeling of the probe (ФW DNA) with digoxigenin-11-UTP was done by random priming (DIG DNA labeling and detection kit, Roche). Hybridization was done under stringent conditions at 68°C. Dig-labeled hybrids were detected by an anti-Dig alkaline phosphatase conjugate and the substrates NBT and BCIP.

### Negative Staining and Electron Microscopy of the Phages

Thin carbon support films were prepared by sublimation of a carbon thread onto a freshly cleaved mica surface. Phages were negatively stained with 4% (w/v) aqueous uranyl acetate, pH 5.0, according to the method of Valentine et al. [76]. Samples were examined in a TEM 910 transmission electron microscope (Carl Zeiss, Oberkochen) at an acceleration voltage of 80 kV. Images were taken at calibrated magnifications using a line replica. Images were recorded digitally with a Slow-Scan CCD-Camera (ProScan, 1024×1024, Scheuring, Germany) with ITEM-Software (Olympus Soft Imaging Solutions, Münster, Germany). Tails and heads of all phages were measured (Table 1).

## Supporting Information

Figure S1 **Molecular phylogenetic analysis by maximum likelihood method of the** ***rec*** **AF gene.** The evolutionary history was inferred by using the maximum likelihood method based on the Jukes-Cantor model. The tree with the highest log likelihood is shown, analyses were conducted in MEGA5.
(TIF)

Figure S2 **eBurst analysis of different sequence types.** The eBURST diagramm depicts the relationship among sequence types. Black lines indicate single locus variants, i.e, one out of four loci as compared between two sequence types is different. Blue lines indicate double locus variants. Sequence types that are not linked by any line differ by at least three out of four loci.
(TIF)

Table S1 **Metadata of all** ***Achromobacter*** **strains of this study.**
(XLSX)

File S1 **Supplemental file describing the details of the phenotype microarray data analysis.**
(PDF)
