# Supplementary figures and images for: Isolation and Characterization of Numerous Novel Phages Targeting Diverse Strains of the Ubiquitous and Opportunistic Pathogen Achromobacter xylosoxidans
Source: PLoS One. 2014 Jan 22;9(1):e86935. doi: 10.1371/journal.pone.0086935 (PMC3899368; doi:10.1371/journal.pone.0086935)

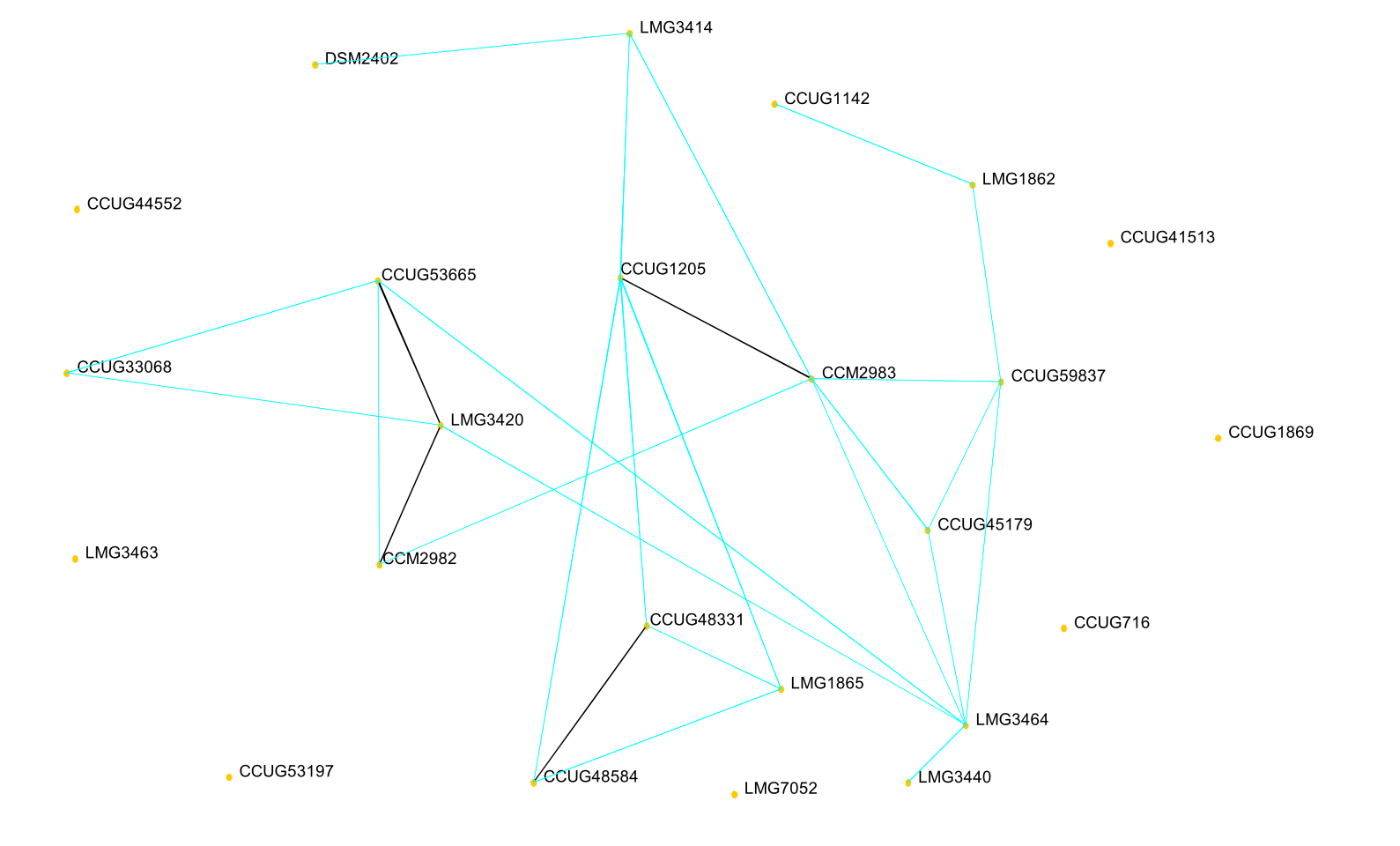

Supplement: Figure S1 — Molecular phylogenetic analysis by maximum likelihood method of the rec AF gene. The evolutionary history was inferred by using the maximum likelihood method based on the Jukes-Cantor model. The tree with the highest log likelihood is shown, analyses were conducted in MEGA5. (TIF) [file pone.0086935.s001.tif]

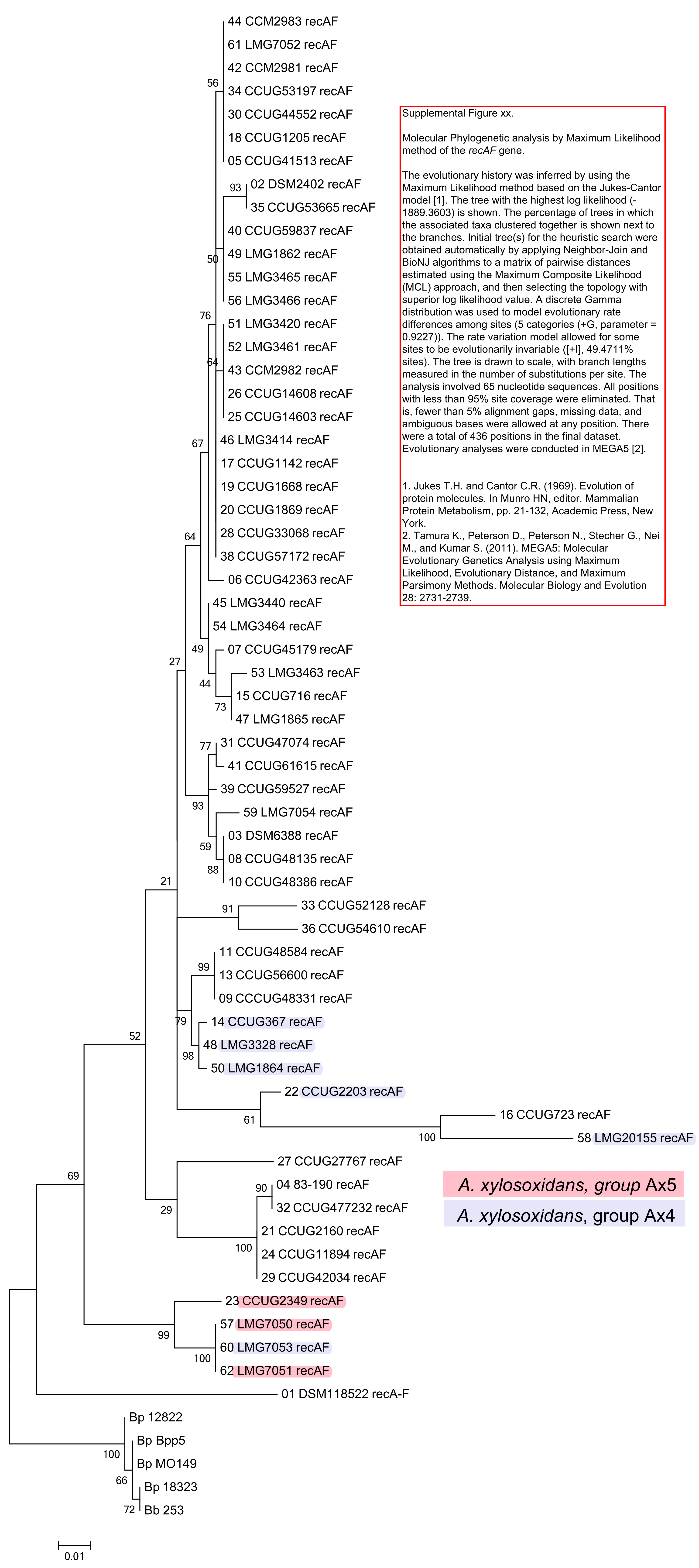

Supplement: Figure S2 — eBurst analysis of different sequence types. The eBURST diagramm depicts the relationship among sequence types. Black lines indicate single locus variants, i.e, one out of four loci as compared between two sequence types is different. Blue lines indicate double locus variants. Sequence types that are not linked by any line differ by at least three out of four loci. (TIF) [file pone.0086935.s002.tif]
