# Supplementary material for: Isolation and Characterization of Numerous Novel Phages Targeting Diverse Strains of the Ubiquitous and Opportunistic Pathogen Achromobacter xylosoxidans
Source: PLoS One. 2014 Jan 22;9(1):e86935. doi: 10.1371/journal.pone.0086935 (PMC3899368; doi:10.1371/journal.pone.0086935)
Supplement: File S1 — Supplemental file describing the details of the phenotype microarray data analysis. (PDF) [file pone.0086935.s004.pdf]

Isolation of numerous novel phages targeting highly antibiotic resistant strains of the pathogen *Achromobacter xylosoxidans* Supplemental Material - Analysis of Phenotype Microarray Gen III plates -

Johannes Wittmann
Leibniz Institute DSMZ

Brigitte Dreiseikelman
University of Bielefeld

Christine Rohde
Leibniz Institute DSMZ

Manfred Rohde
Helmholtz Centre for Infection Research

Johannes Sikorski
Leibniz Institute DSMZ

Abstract

This file describes the analysis of data from OmniLog® Phenotype MicroArray Gen III plates of *Achromobacter xylosoxidans* strains. The file consists of four main parts:

- Presentation of the data structure
- Heatmap analysis of "Area under the curve" (AUC) curve parameter values
- Principal Component Analysis
- Identification of physiological reactions that are significantly different between any of the *A. xylosoxidans* groups Ax1 to Ax6.

The R code is embedded.
This is the version of October 7, 2013.

Keywords: OmniLog® Phenotype MicroArray, heatmap, Principal Component Analysis, multiple comparison of groups.

This is a **Sweave** file (Leisch 2002). **Sweave** is a tool that allows to embed the R code for complete data analyses in LaTeX documents. The purpose is to create dynamic reports, which can be updated automatically if data or analysis change. Instead of inserting a prefabricated graph or table into the report, the master document contains the R code necessary to obtain it. When run through R, all data analysis output (tables, graphs, etc.) is created on the fly and inserted into a final latex document. The report can be automatically updated if data or analysis change, which allows for truly reproducible research.

For details to the code we refer to the tutorial and R manual of the **opm** package. All web resources regarding **opm** are linked on its main website `http://opm.dsmz.de/`. Strategies to analyse OmniLog® data are discussed in (Vaas, Sikorski, Michael, Göker, and Klenk 2012; Vaas, Sikorski, Hofner, Fiebig, Buddruhs, Klenk, and Göker 2013). For further details to the OmniLog® technology we refer to (Bochner, Gadzinski, and Panomitros 2001; Bochner

2009).
Load the neccessary R.

```
R> # general
R> rm(list=ls(all=TRUE))   # removes all functions, libraries, data etc
R> # load packages
R> library(opm)
R> library(RColorBrewer)
R> pal <- brewer.pal(8,"Greens")  # color palette from the brewer package
R> library(BiodiversityR)
R> library(vegan)
```

1. The data

The raw kinetic data of 121 Gen III microplates have been compiled into an `OPMS` object as described in the tutorial of the **opm** package (http://opm.dsmz.de/). The curve parameters have been aggregated using spline-fit algorithms as described (Vaas *et al.* 2013). Additional metadata were included to the dataset. The final dataset is loaded using the code below.

```
R> load("Achromobacter_SOM.RData")
```

Provide a brief overview on the associated metadata.

```
R> flat.achr <- flatten(achr[,1,"A01"],
                        include = list("city","country", "genus",
                                       "habitat","Isolator","replicate","species" ,
                                       "ssource","strain","year", "MLSTcluster"))
```

```
R> options(width=170)
R> flat.achr[, c(1:11)]
```

```
         city    country         genus       habitat         Isolator replicate       species        ssource     strain    year MLSTcluster
1     unknown    Austria Achromobacter          soil      Streichsbier         3  xylosoxidans environmental   DSM11852    1991     Ax_none
2     unknown    Austria Achromobacter          soil      Streichsbier         1  xylosoxidans environmental   DSM11852    1991     Ax_none
3        Wien    Austria Achromobacter        sputum         H. Masoud         1  xylosoxidans       medical CCUG 41513    1998         Ax1
4        Wien    Austria Achromobacter        sputum  A. Makristathis         1  xylosoxidans       medical CCUG 45179    2001         Ax1
5       Paris     France Achromobacter       unknown Vieu und Binette         1  xylosoxidans       unknown     83-190    1983         Ax6
6     unknown    Austria Achromobacter          soil      Streichsbier         2  xylosoxidans environmental   DSM11852    1991     Ax_none
7        Wien    Austria Achromobacter        sputum         H. Masoud         2  xylosoxidans       medical CCUG 41513    1998         Ax1
8        Wien    Austria Achromobacter        sputum  A. Makristathis         2  xylosoxidans       medical CCUG 45179    2001         Ax1
9       Paris     France Achromobacter       unknown Vieu und Binette         2  xylosoxidans       unknown     83-190    1983         Ax6
10  Goeteborg     Sweden Achromobacter ear discharge           unknown         1  xylosoxidans       medical  CCUG 1142    1971         Ax1
11       Lyon     France Achromobacter         water      J. Fleurette         1  xylosoxidans environmental CCUG 14603    1983         Ax1
12       Lyon     France Achromobacter    nosocomial      J. Fleurette         1  xylosoxidans       medical CCUG 14608    1983         Ax1
13    unknown    Germany Achromobacter         wound           unknown         1  xylosoxidans       medical  CCUG 2160 unknown         Ax6
14  Goeteborg     Sweden Achromobacter ear discharge           unknown         1  xylosoxidans       medical  CCUG 2203    1973         Ax4
15 Manchester    England Achromobacter         blood           unknown         1     ruhlandii       medical  CCUG 2349    1950         Ax5
16    unknown    unknown Achromobacter       unknown      F. Pichinoty         1  xylosoxidans       unknown   CCM 2981 unknown         Ax1
17    unknown    unknown Achromobacter       unknown      F. Pichinoty         1  xylosoxidans       unknown   CCM 2982 unknown         Ax1
18  Goeteborg     Sweden Achromobacter         urine           unknown         1  xylosoxidans       medical CCUG 42034    1999         Ax6
19  Goeteborg     Sweden Achromobacter        sputum           unknown         1  xylosoxidans       medical CCUG 42363    1999         Ax1
20  Goeteborg     Sweden Achromobacter        sputum           unknown         1  xylosoxidans       medical CCUG 47074    2002         Ax2
21     Dublin    Ireland Achromobacter       unknown           unknown         1            sp       medical CCUG 47723    2003         Ax6
22  Goeteborg     Sweden Achromobacter        sputum       B. Joensson         1  xylosoxidans       medical CCUG 48331    2003         Ax1
23  Goeteborg     Sweden Achromobacter        sputum         Lena Lind         1  xylosoxidans       medical CCUG 48684    2004         Ax1
24  Goeteborg     Sweden Achromobacter        sputum           unknown         1  xylosoxidans       medical CCUG 53197    2006         Ax1
25  Goeteborg     Sweden Achromobacter           eye           unknown         1  xylosoxidans       medical CCUG 54610    2007         Ax3
26  Goeteborg     Sweden Achromobacter        sputum        Adlerberth         1  xylosoxidans       medical CCUG 56600    2008         Ax1
27  Goeteborg     Sweden Achromobacter        sputum           unknown         1  xylosoxidans       medical CCUG 57172    2008         Ax1
28  Goeteborg     Sweden Achromobacter        sputum           unknown         1  xylosoxidans       medical CCUG 59527    2010         Ax2
29  Goeteborg     Sweden Achromobacter         blood           unknown         1  xylosoxidans       medical CCUG 61615    2011         Ax2
30  Goeteborg     Sweden Achromobacter ear discharge           unknown         2  xylosoxidans       medical  CCUG 1142    1971         Ax1
31       Lyon     France Achromobacter         water      J. Fleurette         2  xylosoxidans environmental CCUG 14603    1983         Ax1
32       Lyon     France Achromobacter    nosocomial      J. Fleurette         2  xylosoxidans       medical CCUG 14608    1983         Ax1
33    unknown    Germany Achromobacter         wound           unknown         2  xylosoxidans       medical  CCUG 2160 unknown         Ax6
34  Goeteborg     Sweden Achromobacter ear discharge           unknown         2  xylosoxidans       medical  CCUG 2203    1973         Ax4
35 Manchester    England Achromobacter         blood           unknown         2     ruhlandii       medical  CCUG 2349    1950         Ax5
36    unknown    unknown Achromobacter       unknown      F. Pichinoty         2  xylosoxidans       unknown   CCM 2981 unknown         Ax1
37    unknown    unknown Achromobacter       unknown      F. Pichinoty         2  xylosoxidans       unknown   CCM 2982 unknown         Ax1
38  Goeteborg     Sweden Achromobacter         urine           unknown         2  xylosoxidans       medical CCUG 42034    1999         Ax6
```

39	Goeteborg	Sweden	Achromobacter	sputum	unknown	2	xylosoxidans	medical	CCUG 42363	1999	Ax1
40	Goeteborg	Sweden	Achromobacter	sputum	unknown	2	xylosoxidans	medical	CCUG 47074	2002	Ax2
41	Dublin	Ireland	Achromobacter	unknown	unknown	2	sp	medical	CCUG 47723	2003	Ax6
42	Goeteborg	Sweden	Achromobacter	sputum	B. Joensson	2	xylosoxidans	medical	CCUG 48331	2003	Ax1
43	Goeteborg	Sweden	Achromobacter	sputum	Lena Lind	2	xylosoxidans	medical	CCUG 48684	2004	Ax1
44	Goeteborg	Sweden	Achromobacter	sputum	unknown	2	xylosoxidans	medical	CCUG 53197	2006	Ax1
45	Goeteborg	Sweden	Achromobacter	eye	unknown	2	xylosoxidans	medical	CCUG 54610	2007	Ax3
46	Goeteborg	Sweden	Achromobacter	sputum	Adlerberth	2	xylosoxidans	medical	CCUG 56600	2008	Ax1
47	Goeteborg	Sweden	Achromobacter	sputum	unknown	2	xylosoxidans	medical	CCUG 57172	2008	Ax1
48	Goeteborg	Sweden	Achromobacter	sputum	unknown	2	xylosoxidans	medical	CCUG 59527	2010	Ax2
49	Goeteborg	Sweden	Achromobacter	blood	unknown	2	xylosoxidans	medical	CCUG 61615	2011	Ax2
50	Goeteborg	Sweden	Achromobacter	blood	unknown	1	xylosoxidans	medical	CCUG 11894	1982	Ax6
51	unknown	Spain	Achromobacter	spinal fluid	unknown	1	xylosoxidans	medical	CCUG 1205	1970	Ax1
52	Goeteborg	Sweden	Achromobacter	ear discharge	unknown	1	xylosoxidans	medical	CCUG 1668	1972	Ax1
53	unknown	unknown	Achromobacter	unknown	unknown	1	xylosoxidans	unknown	CCUG 1869	1972	Ax1
54	unknown	Japan	Achromobacter	ear discharge	Eiko Yabuuchi	1	xylosoxidans	medical	DSM2402	unknown	Ax_none
55	Stockholm	Sweden	Achromobacter	unknown	unknown	1	denitrificans	unknown	CCUG 27767	1991	Ax_none
56	unknown	France	Achromobacter	urine	F. Pichinoty	1	xylosoxidans	medical	CCM 2983	unknown	Ax1
57	Falun	Sweden	Achromobacter	sputum	unknown	1	xylosoxidans	medical	CCUG 33068	1994	Ax1
58	Copenhagen	Denmark	Achromobacter	unknown	unknown	1	xylosoxidans	unknown	CCUG 367	1964	Ax4
59	Goeteborg	Sweden	Achromobacter	sputum	unknown	1	xylosoxidans	medical	CCUG 44552	2000	Ax1
60	Goeteborg	Sweden	Achromobacter	sputum	B. Joensson	1	xylosoxidans	medical	CCUG 48135	2003	Ax2
61	Goeteborg	Sweden	Achromobacter	sputum	C. Ahren	1	xylosoxidans	medical	CCUG 48386	2003	Ax2
62	Goeteborg	Sweden	Achromobacter	sputum	C. Wenneras	1	xylosoxidans	medical	CCUG 48584	2003	Ax1
63	unknown	Sweden	Achromobacter	unknown	unknown	1	xylosoxidans	environmental	CCUG 52128	2005	Ax3
64	Goeteborg	Sweden	Achromobacter	sputum	unknown	1	xylosoxidans	medical	CCUG 53665	2006	Ax1
65	Goeteborg	Sweden	Achromobacter	sputum	unknown	1	xylosoxidans	medical	CCUG 56295	2008	Ax_none
66	Goeteborg	Sweden	Achromobacter	trachea	unknown	1	xylosoxidans	medical	CCUG 59837	2010	Ax1
67	Goettingen	Germany	Achromobacter	unknown	Nagel	1	xylosoxidans	unknown	DSM6388	unknown	Ax2
68	Goeteborg	Sweden	Achromobacter	unknown	unknown	1	xylosoxidans	unknown	CCUG 716	1970	Ax1
69	Paris	France	Achromobacter	unknown	unknown	1	xylosoxidans	unknown	CCUG 723	1961	Ax1
70	Goeteborg	Sweden	Achromobacter	blood	unknown	2	xylosoxidans	medical	CCUG 11894	1982	Ax6
71	unknown	Spain	Achromobacter	spinal fluid	unknown	2	xylosoxidans	medical	CCUG 1205	1970	Ax1
72	Lyon	France	Achromobacter	water	J. Fleurette	2	xylosoxidans	environmental	CCUG 14603	1983	Ax1
73	Goeteborg	Sweden	Achromobacter	ear discharge	unknown	2	xylosoxidans	medical	CCUG 1668	1972	Ax1
74	unknown	unknown	Achromobacter	unknown	unknown	2	xylosoxidans	unknown	CCUG 1869	1972	Ax1
75	Stockholm	Sweden	Achromobacter	unknown	unknown	2	denitrificans	unknown	CCUG 27767	1991	Ax_none
76	unknown	France	Achromobacter	urine	F. Pichinoty	2	xylosoxidans	medical	CCM 2983	unknown	Ax1
77	Falun	Sweden	Achromobacter	sputum	unknown	2	xylosoxidans	medical	CCUG 33068	1994	Ax1
78	Copenhagen	Denmark	Achromobacter	unknown	unknown	2	xylosoxidans	unknown	CCUG 367	1964	Ax4
79	Goeteborg	Sweden	Achromobacter	sputum	unknown	2	xylosoxidans	medical	CCUG 44552	2000	Ax1
80	Goeteborg	Sweden	Achromobacter	sputum	B. Joensson	2	xylosoxidans	medical	CCUG 48135	2003	Ax2
81	Goeteborg	Sweden	Achromobacter	sputum	C. Ahren	2	xylosoxidans	medical	CCUG 48386	2003	Ax2
82	Goeteborg	Sweden	Achromobacter	sputum	C. Wenneras	2	xylosoxidans	medical	CCUG 48584	2003	Ax1
83	unknown	Sweden	Achromobacter	unknown	unknown	2	xylosoxidans	environmental	CCUG 52128	2005	Ax3
84	Goeteborg	Sweden	Achromobacter	sputum	unknown	2	xylosoxidans	medical	CCUG 53665	2006	Ax1
85	Goeteborg	Sweden	Achromobacter	sputum	unknown	2	xylosoxidans	medical	CCUG 56295	2008	Ax_none
86	Goeteborg	Sweden	Achromobacter	trachea	unknown	2	xylosoxidans	medical	CCUG 59837	2010	Ax1
87	Goettingen	Germany	Achromobacter	unknown	Nagel	2	xylosoxidans	unknown	DSM6388	unknown	Ax2
88	Goeteborg	Sweden	Achromobacter	unknown	unknown	2	xylosoxidans	unknown	CCUG 716	1970	Ax1
89	Paris	France	Achromobacter	unknown	unknown	2	xylosoxidans	unknown	CCUG 723	1961	Ax1
90	unknown	Denmark	Achromobacter	spinal fluid	unknown	1	xylosoxidans	medical	LMG 1862	unknown	Ax1
91	unknown	Japan	Achromobacter	ear discharge	E. Yabuuchi	1	ruhlandii	medical	LMG 1864	unknown	Ax4
92	unknown	Japan	Achromobacter	soil	Iwasaki and Mori	1	xylosoxidans	environmental	LMG 1865	unknown	Ax1
93	Jalajala	Philippines	Achromobacter	unknown	B.Cottyn	1	xylosoxidans	environmental	LMG 20155	unknown	Ax4
94	unknown	France	Achromobacter	blood	unknown	1	ruhlandii	medical	LMG 3328	unknown	Ax4
95	unknown	unknown	Achromobacter	unknown	unknown	1	denitrificans	unknown	LMG 3414	unknown	Ax1
96	unknown	Japan	Achromobacter	ear discharge	E. Yabuuchi	1	xylosoxidans	medical	LMG 3420	unknown	Ax1
97	unknown	France	Achromobacter	sputum	unknown	1	xylosoxidans	medical	LMG 3440	unknown	Ax1
98	unknown	Denmark	Achromobacter	ear discharge	H. Lautrop	1	xylosoxidans	medical	LMG 3461	unknown	Ax1
99	unknown	Denmark	Achromobacter	spinal fluid	H. Lautrop	1	xylosoxidans	medical	LMG 3463	unknown	Ax1
100	unknown	Denmark	Achromobacter	water	H. Lautrop	1	xylosoxidans	environmental	LMG 3464	unknown	Ax1
101	unknown	Denmark	Achromobacter	water	H. Lautrop	1	xylosoxidans	environmental	LMG 3465	unknown	Ax1
102	unknown	Denmark	Achromobacter	unknown	H. Lautrop	1	xylosoxidans	medical	LMG 3466	unknown	Ax1
103	unknown	Wales	Achromobacter	blood	unknown	1	xylosoxidans	medical	LMG 7050	unknown	Ax5
104	unknown	England	Achromobacter	blood	unknown	1	xylosoxidans	medical	LMG 7051	unknown	Ax5
105	unknown	England	Achromobacter	sputum	unknown	1	xylosoxidans	medical	LMG 7052	unknown	Ax1
106	unknown	Scotland	Achromobacter	wound	unknown	1	xylosoxidans	medical	LMG 7053	unknown	Ax4
107	unknown	England	Achromobacter	unknown	unknown	1	xylosoxidans	environmental	LMG 7054	unknown	Ax2
108	unknown	Japan	Achromobacter	ear discharge	Eiko Yabuuchi	1	xylosoxidans	medical	DSM2402	unknown	Ax_none
109	unknown	France	Achromobacter	urine	F. Pichinoty	2	xylosoxidans	medical	CCM 2983	unknown	Ax1
110	unknown	Denmark	Achromobacter	spinal fluid	unknown	2	xylosoxidans	medical	LMG 1862	unknown	Ax1
111	unknown	Japan	Achromobacter	ear discharge	E. Yabuuchi	2	ruhlandii	medical	LMG 1864	unknown	Ax4
112	unknown	Japan	Achromobacter	soil	Iwasaki and Mori	2	xylosoxidans	environmental	LMG 1865	unknown	Ax1
113	Jalajala	Philippines	Achromobacter	unknown	B.Cottyn	2	xylosoxidans	environmental	LMG 20155	unknown	Ax4
114	unknown	France	Achromobacter	blood	unknown	2	ruhlandii	medical	LMG 3328	unknown	Ax4
115	unknown	unknown	Achromobacter	unknown	unknown	2	denitrificans	unknown	LMG 3414	unknown	Ax1
116	unknown	Japan	Achromobacter	ear discharge	E. Yabuuchi	2	xylosoxidans	medical	LMG 3420	unknown	Ax1
117	unknown	France	Achromobacter	sputum	unknown	2	xylosoxidans	medical	LMG 3440	unknown	Ax1
118	unknown	Denmark	Achromobacter	ear discharge	H. Lautrop	2	xylosoxidans	medical	LMG 3461	unknown	Ax1
119	unknown	Denmark	Achromobacter	spinal fluid	H. Lautrop	2	xylosoxidans	medical	LMG 3463	unknown	Ax1
120	unknown	Denmark	Achromobacter	water	H. Lautrop	2	xylosoxidans	environmental	LMG 3464	unknown	Ax1
121	unknown	Denmark	Achromobacter	water	H. Lautrop	2	xylosoxidans	environmental	LMG 3465	unknown	Ax1
122	unknown	Denmark	Achromobacter	unknown	H. Lautrop	2	xylosoxidans	medical	LMG 3466	unknown	Ax1
123	unknown	Wales	Achromobacter	blood	unknown	2	xylosoxidans	medical	LMG 7050	unknown	Ax5
124	unknown	England	Achromobacter	blood	unknown	2	xylosoxidans	medical	LMG 7051	unknown	Ax5
125	unknown	England	Achromobacter	sputum	unknown	2	xylosoxidans	medical	LMG 7052	unknown	Ax1
126	unknown	Scotland	Achromobacter	wound	unknown	2	xylosoxidans	medical	LMG 7053	unknown	Ax4
127	unknown	England	Achromobacter	unknown	unknown	2	xylosoxidans	environmental	LMG 7054	unknown	Ax2

2. Heatmap on AUC values

The curve kinetics will be analysed using the curve parameter ”Area under the curve” (AUC). A heatmap analysis allows to cluster strains according to similar physiological reactions across all 96 wells. Similarly, wells are clustered according to similarities across all strains.
The code below shows Figure 1.

```
R> heat_map(achromobacter,
                  as.labels = list("strain", "replicate", "MLSTcluster"),   # labels of the rows
                  as.groups = "MLSTcluster",      # colors the rowside dendrogram
                  cexRow = 0.5,            # set the size of the row labels
                  use.fun = "gplots",
                  main = "Heatmap on AUC data",
                  subset  =  "AUC",
                  r.col = c("white", "palegreen1", "khaki1", "grey74",
                           "lightblue1", "lightpink", "lightsalmon1"),
                  col = pal,
                  xlab = "Well substrates on GenIII Biolog plate",
                  ylab = "strains, replicates, and their MLST cluster affiliation")
```

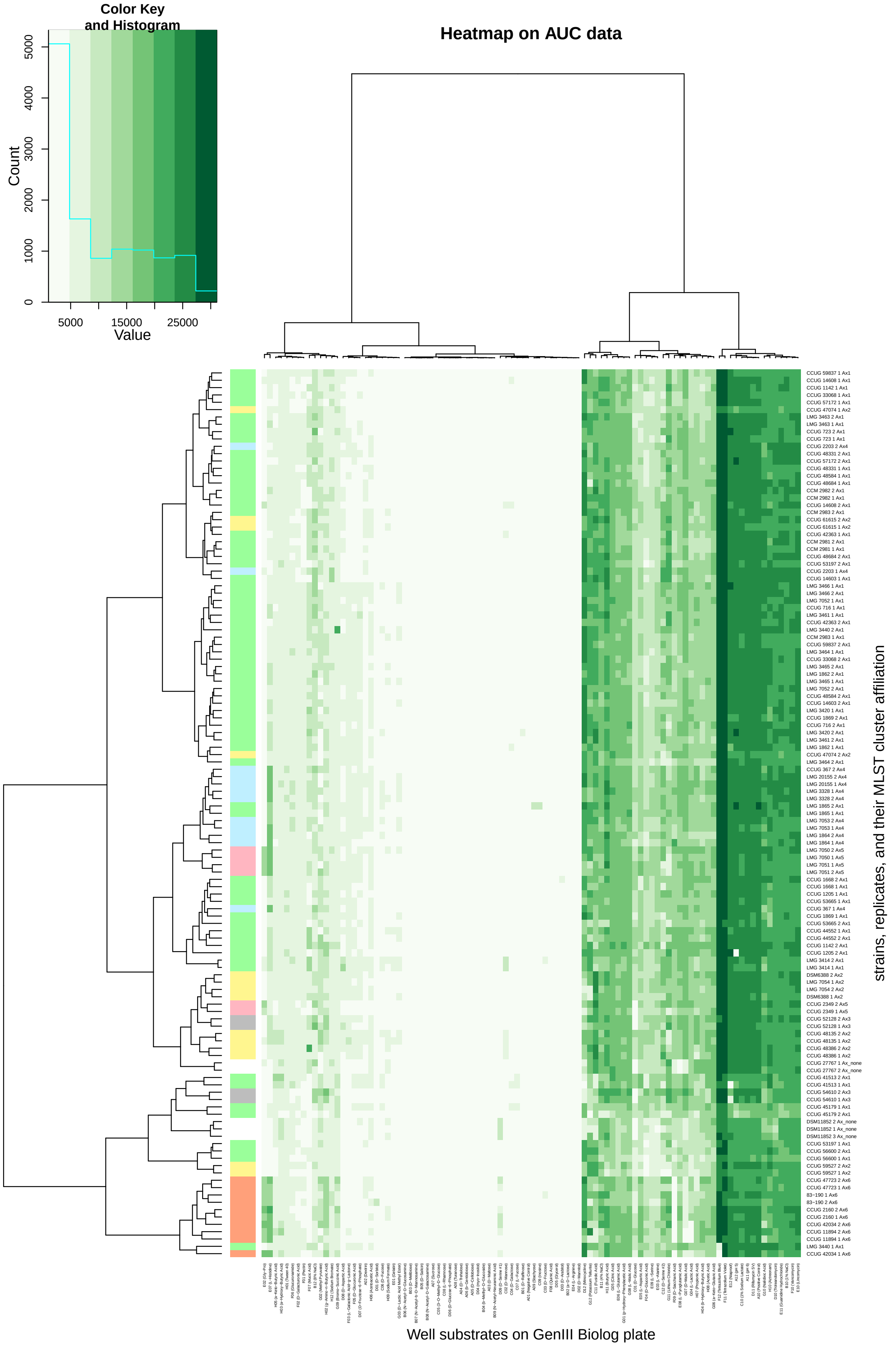

Figure 1: Gen III heatmap of the curve parameter "AUC", which is the area under the curve. The row dendrogramm color follows MLST cluster designation (see also the last entry in the row labels). The dendrograms are created using ward clustering on euclidian distances.

3. Principal Component Analysis of GenIII plates

Principal Component Analysis (PCA) is a mathematical procedure that uses orthogonal transformation to convert a set of observations of possibly correlated variables into a set of values of linearly uncorrelated variables called principal components (http://en.wikipedia.org/wiki/Principal_component_analysis, access on October 7, 2013). The idea is to detect and plot only the actually existing relationships between the variables. More technical and mathematically precise definitions are given in (Legendre and Legendre 1998).

Load custom principal component analysis function. This function makes use of the R packages **vegan** and **BiodiversityR**.

```
R> custom_pca <- function(x, legend = FALSE, lwd = 2, text.col = "darkgrey",
      arrow.col = "red", cex = 0.4, scaling = 1, circle = scaling == 1,
      pre.sym.col = if (symbols)
        "white" # avoid plotting ordisymbol() symbols over biplot() symbols
      else
        "black", symbols = TRUE, ...) {
    pca <- vegan::rda(x)
    #print(summary(pca)[6])
    pca.plot <- biplot(x = pca, scaling = scaling,
      col = c(pre.sym.col, arrow.col), ...)
    if (symbols)
      BiodiversityR::ordisymbol(pca.plot,
        data.frame(.N = if (is.null(attr(x, "row.groups")))
          rownames(x)
        else
          attr(x, "row.groups")), ".N", legend = legend, lwd = lwd)
    text(pca.plot, "species", col = text.col, cex = cex)
    if (circle)
      BiodiversityR::ordiequilibriumcircle(pca, pca.plot)
    invisible(pca)
  }
```

Prepare a data matrix for a principal component analysis based on curve parameter "AUC".

```
R> x <- opm::extract(achromobacter, as.labels = list("strain", "replicate", "MLSTcluster"),
    as.groups = list("MLSTcluster"), subset = "AUC")
```

The code below shows the pca ordination Figure 2.

```
R> custom_pca(x, legend = TRUE, cex = 0.3, text.col = "grey")
```

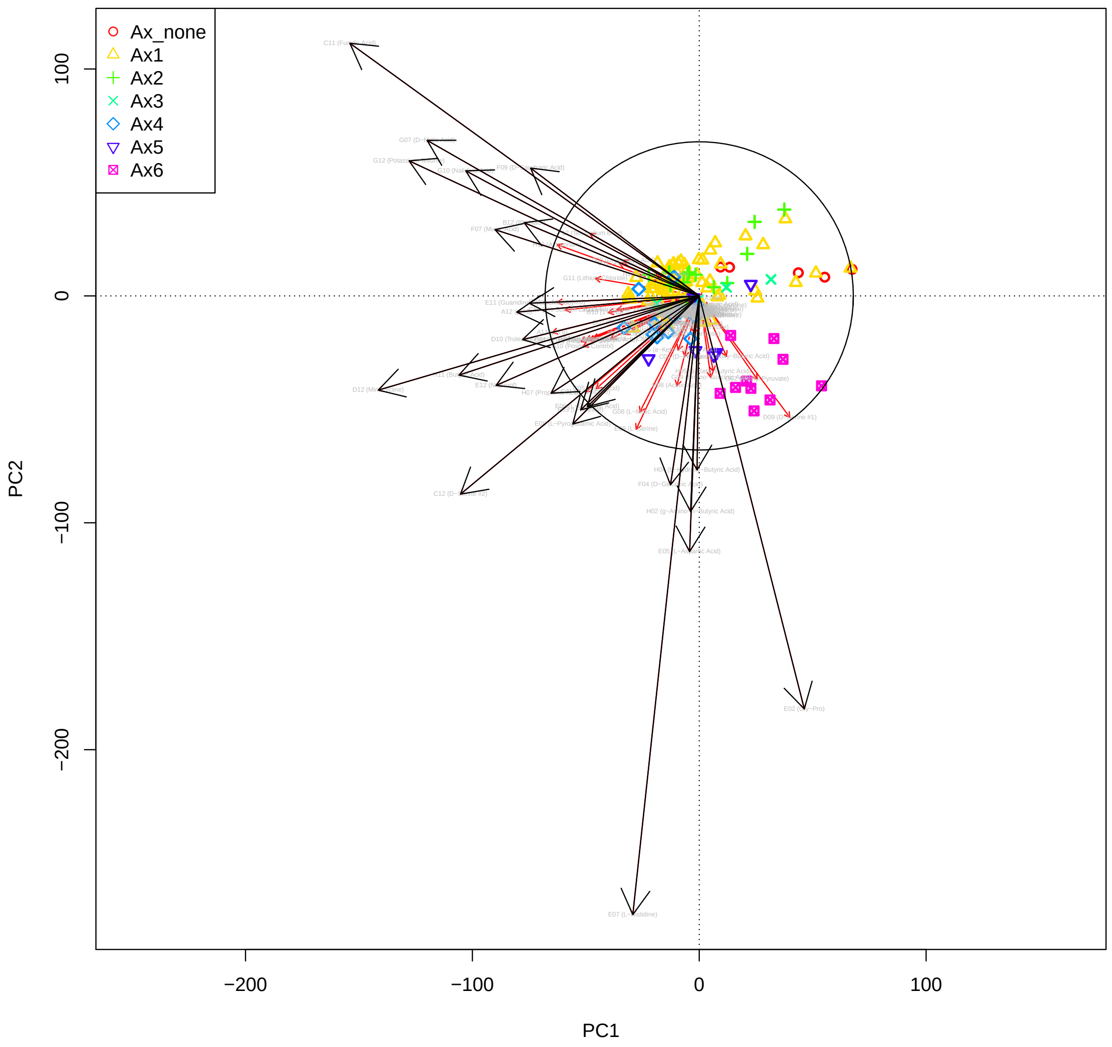

Figure 2: Biplot graphical representation of a principal component analysis of curve parameter "AUC" values as shown in the heatmap of Figure 1. The axes PC1 and PC2 display 24.1% and 13.9% of the total variance, respectively. Function `ordiequilibriumcircle()` produces a *circle of equilibrium contribution*. Its radius is equal to $\sqrt{d/p}$, where d is the number of axes represented in the biplot (usually $d = 2$) and p is the number of dimensions of the PCA space (i.e. usually the number of variables of the data matrix, here with $N = 96$). The radius of this circle represents the length of the vector representing a variable that would contribute equally to all the dimensions of the PCA space. Therefore, for any given pair of axes, the variables that have vectors longer than this radius (in black color) make a higher contribution than average and can be interpreted with confidence (Borcard *et al.* 2011), whereas variables that are not informative are represented by red vectors. The figure demonstrates that numerous physiological reactions (represented by the wells) contribute to the PCA space. In general, strains of individual groups Ax1 to Ax2 occupy similar positions in the PCA place, which supports the results from the heatmap analysis (Figure 1).

4. Multiple Comparison per Well

A further research task is to identify whether among any of the six phylogenetically defined groups Ax1 to Ax6 of *A. xylosoxidans* (see Fig. 1 of the main manuscript) significant differences in the AUC values of any of the 96 wells are. A "Tukey"-type of comparison (all-against-all) of the groups Ax1 to Ax6 would result in 15 pairwise comparisons. Hence, this results in 15 statistical hypotheses that are tested simultaneously. If an increasing number of hypotheses is tested, with the number of true hypotheses unknown, the probability of at least one wrong testing decision also increases. That is, conclusions on signifcant differences between a pair of groups are increasingly likely to be wrong. Thus the so-called family-wise error-rate (or False Discovery Rate), which is essentially the probability of at least one false rejection among all the null hypotheses, needs to be controlled. The function `opm_mcp()` applies functionality from the **multcomp** package which has appropriate control of the family-wise error-rate implemented ((Hothorn, Bretz, and Westfall 2008; Herberich, Sikorski, and Hothorn 2010)).

Therefore, in order to identify between which of the six Ax groups (see Figure 1 of the main text) a significant difference exists, a multiple comparison of Ax groups per well is performed using `multcomp` algorithms ((Hothorn *et al.* 2008; Herberich *et al.* 2010)).
Prepare the data set for application of `opm_mcp()` per well. Remove the Ax_none strains.

```
R> achr1 <- subset(achromobacter, query = list(MLSTcluster = "Ax_none"), invert = TRUE)
R> allpvalues <- c()
R> allsubstrate <- c()
R> allestimate <- c()
R> allstrains <- c()
R> specificWell <- opm_mcp(achr1[,,"H02"], model = ~ MLSTcluster,
                          m.type = "aov", linfct = c(Tukey = 1), subset = "AUC")
R> plot(specificWell)
R> strains <- as.vector(row.names(as.data.frame(summary(specificWell)[2])))
```

Perform a multiple comparison among all Ax clusters for each well. These are 15 pairwise comparisons. The comparison is done on the curve parameter "AUC".

```
R> for(i in 1:96){

     specificWell <- opm_mcp(achr1[,,i], model = ~ MLSTcluster,
                          m.type = "aov", linfct = c(Tukey = 1), subset = "AUC")

     # summarize the statistics
     pvalues <- as.vector(unlist(unlist(summary(specificWell)[10])[48:62]))
     estimate <- as.vector(unlist(unlist(summary(specificWell)[10])[3:17]))
     substrate <- rep(wells(achr1[,,i], full = TRUE), 15)
     allpvalues <- c(allpvalues, pvalues)
     allestimate <- c(allestimate, estimate)
     allstrains <- c(allstrains, strains)
     allsubstrate <- c(allsubstrate, substrate)
   }
R> sumpvalues <- data.frame(allstrains, allpvalues,allsubstrate, allestimate)
R> sumpvalues[1276:1290,1:4]
R> sumpvalues$significant.pvalues <- sumpvalues$allpvalues < 0.05
R> sumpvalues$strainA.is.larger.than.strainB <- sumpvalues$allestimate > 0
R> sumpvaluesSIGN <- subset(sumpvalues, significant.pvalues == TRUE)
R> sumpvaluesSIGN$allsubstrate <- factor(sumpvaluesSIGN$allsubstrate)
R> colnames(sumpvaluesSIGN)[1] <- "comparison"
R> colnames(sumpvaluesSIGN)[2] <- "p value"
R> colnames(sumpvaluesSIGN)[3] <- "well and substrate"
R> colnames(sumpvaluesSIGN)[4] <- "difference between AUC means"
```

In which carbon sources is there at least one significant difference between any of the groups Ax1 to Ax6?

```
R> options(width = 80)
R> levels(as.factor(sumpvaluesSIGN[, 3]))
```

```
 [1] "B02 (a-D-Lactose)"                "B12 (8% NaCl)"
 [3] "C01 (D-Glucose)"                  "C05 (3-O-Methyl-D-Glucose)"
 [5] "C10 (1% Sodium Lactate)"          "C11 (Fusidic Acid)"
 [7] "D07 (D-Fructose-6-Phosphate)"     "D08 (D-Aspartic Acid)"
 [9] "D09 (D-Serine)"                   "D12 (Minocycline)"
[11] "E02 (Gly-Pro)"                    "E03 (L-Alanine)"
[13] "E05 (L-Aspartic Acid)"            "E06 (L-Glutamic Acid)"
[15] "E07 (L-Histidine)"                "E09 (L-Serine)"
[17] "E11 (Guanidine Hydrochloride)"    "E12 (Niaproof)"
[19] "F01 (Pectin)"                     "F02 (D-Galacturonic Acid)"
[21] "F03 (L-Galactonic Acid-g-Lactone)" "F04 (D-Gluconic Acid)"
[23] "F05 (D-Glucuronic Acid)"          "F06 (Glucuronamide)"
[25] "F07 (Mucic Acid)"                 "F09 (D-Saccharic Acid)"
[27] "F11 (Tetrazolium Violet)"         "G03 (D-Lactic Acid Methyl Ester)"
[29] "G04 (L-Lactic Acid)"              "G07 (D-Malic Acid)"
[31] "G09 (Bromo-Succinic Acid)"        "G10 (Nalidixic Acid)"
[33] "G11 (Lithium Chloride)"           "G12 (Potassium Tellurite)"
[35] "H01 (Tween 40)"                   "H02 (g-Amino-n-Butyric Acid)"
[37] "H04 (b-Hydroxy-Butyric Acid)"     "H10 (Aztreonam)"
```

List all the significant pairwise comparisons.

```
R> options(width = 150)
R> sumpvaluesSIGN[, 1:4]
```

```
    comparison      p value              well and substrate difference between AUC means
198  Ax4 - Ax1 2.923198e-02               B02 (a-D-Lactose)                     329.9635
347  Ax3 - Ax1 9.024754e-05                   B12 (8% NaCl)                    6043.7674
350  Ax6 - Ax1 7.106421e-03                   B12 (8% NaCl)                   -2968.0389
351  Ax3 - Ax2 1.109162e-04                   B12 (8% NaCl)                    6617.2634
355  Ax4 - Ax3 1.796867e-02                   B12 (8% NaCl)                   -4651.6250
356  Ax5 - Ax3 3.520502e-04                   B12 (8% NaCl)                   -7050.6563
357  Ax6 - Ax3 1.603838e-07                   B12 (8% NaCl)                   -9011.8063
359  Ax6 - Ax4 1.029595e-03                   B12 (8% NaCl)                   -4360.1812
362  Ax3 - Ax1 2.740308e-11                 C01 (D-Glucose)                   -7719.5035
366  Ax3 - Ax2 5.076765e-07                 C01 (D-Glucose)                   -6838.6161
370  Ax4 - Ax3 7.876512e-10                 C01 (D-Glucose)                    7964.6563
371  Ax5 - Ax3 6.157215e-05                 C01 (D-Glucose)                    6105.9375
372  Ax6 - Ax3 5.841532e-06                 C01 (D-Glucose)                    6220.6750
430  Ax4 - Ax3 9.317752e-03      C05 (3-O-Methyl-D-Glucose)                     897.1146
509  Ax6 - Ax4 2.099306e-02         C10 (1% Sodium Lactate)                   -2209.8812
511  Ax2 - Ax1 2.624268e-03              C11 (Fusidic Acid)                    4059.9777
515  Ax6 - Ax1 2.566049e-06              C11 (Fusidic Acid)                   -6928.9187
516  Ax3 - Ax2 1.436754e-02              C11 (Fusidic Acid)                   -6808.3214
519  Ax6 - Ax2 1.721625e-10              C11 (Fusidic Acid)                  -10988.8964
524  Ax6 - Ax4 7.321234e-05              C11 (Fusidic Acid)                   -7376.3667
525  Ax6 - Ax5 2.498316e-03              C11 (Fusidic Acid)                   -7190.7208
635  Ax6 - Ax1 1.915129e-02    D07 (D-Fructose-6-Phosphate)                   -1194.0969
644  Ax6 - Ax4 2.143343e-03    D07 (D-Fructose-6-Phosphate)                   -1836.6958
650  Ax6 - Ax1 3.185582e-05           D08 (D-Aspartic Acid)                   -3151.1521
654  Ax6 - Ax2 1.071763e-03           D08 (D-Aspartic Acid)                   -3146.9661
657  Ax6 - Ax3 4.562262e-02           D08 (D-Aspartic Acid)                   -3195.7562
665  Ax6 - Ax1 0.000000e+00                  D09 (D-Serine)                    4292.0667
669  Ax6 - Ax2 0.000000e+00                  D09 (D-Serine)                    4378.7482
672  Ax6 - Ax3 1.265654e-14                  D09 (D-Serine)                    4494.7125
674  Ax6 - Ax4 0.000000e+00                  D09 (D-Serine)                    4145.6396
675  Ax6 - Ax5 2.220446e-16                  D09 (D-Serine)                    4290.9208
```

706	Ax2 - Ax1	1.141524e-06	D12 (Minocycline)	-6833.9085
707	Ax3 - Ax1	5.284286e-08	D12 (Minocycline)	-13077.8281
709	Ax5 - Ax1	1.277779e-03	D12 (Minocycline)	-6939.8073
712	Ax4 - Ax2	6.241677e-04	D12 (Minocycline)	6796.2470
714	Ax6 - Ax2	1.482354e-03	D12 (Minocycline)	6729.5679
715	Ax4 - Ax3	2.342226e-06	D12 (Minocycline)	13040.1667
717	Ax6 - Ax3	1.491997e-05	D12 (Minocycline)	12973.4875
718	Ax5 - Ax4	1.078187e-02	D12 (Minocycline)	-6902.1458
720	Ax6 - Ax5	1.669333e-02	D12 (Minocycline)	6835.4667
739	Ax5 - Ax1	0.000000e+00	E02 (Gly-Pro)	9977.0069
740	Ax6 - Ax1	0.000000e+00	E02 (Gly-Pro)	11289.9444
743	Ax5 - Ax2	0.000000e+00	E02 (Gly-Pro)	9892.0536
744	Ax6 - Ax2	0.000000e+00	E02 (Gly-Pro)	11204.9911
746	Ax5 - Ax3	0.000000e+00	E02 (Gly-Pro)	10179.7187
747	Ax6 - Ax3	0.000000e+00	E02 (Gly-Pro)	11492.6562
748	Ax5 - Ax4	0.000000e+00	E02 (Gly-Pro)	9383.7604
749	Ax6 - Ax4	0.000000e+00	E02 (Gly-Pro)	10696.6979
752	Ax3 - Ax1	7.325594e-03	E03 (L-Alanine)	3203.0799
756	Ax3 - Ax2	3.488617e-04	E03 (L-Alanine)	4434.0446
762	Ax6 - Ax3	2.164407e-04	E03 (L-Alanine)	-4731.6250
765	Ax6 - Ax5	3.956553e-02	E03 (L-Alanine)	-2699.4583
785	Ax6 - Ax1	1.105835e-04	E05 (L-Aspartic Acid)	3735.6899
789	Ax6 - Ax2	1.295726e-02	E05 (L-Aspartic Acid)	3277.9268
792	Ax6 - Ax3	9.084223e-03	E05 (L-Aspartic Acid)	4848.1812
794	Ax6 - Ax4	2.105298e-02	E05 (L-Aspartic Acid)	3229.5146
801	Ax3 - Ax2	1.488507e-03	E06 (L-Glutamic Acid)	3765.1563
802	Ax4 - Ax2	3.224592e-02	E06 (L-Glutamic Acid)	1983.4167
807	Ax6 - Ax3	1.549068e-02	E06 (L-Glutamic Acid)	-3234.2938
813	Ax4 - Ax1	1.180172e-09	E07 (L-Histidine)	7450.7622
814	Ax5 - Ax1	4.742383e-04	E07 (L-Histidine)	6282.5851
815	Ax6 - Ax1	9.888756e-13	E07 (L-Histidine)	9536.9101
817	Ax4 - Ax2	1.296690e-09	E07 (L-Histidine)	9339.7307
818	Ax5 - Ax2	3.209675e-05	E07 (L-Histidine)	8171.5536
819	Ax6 - Ax2	2.877809e-12	E07 (L-Histidine)	11425.8786
820	Ax4 - Ax3	1.598760e-03	E07 (L-Histidine)	7790.9271
821	Ax5 - Ax3	3.394770e-02	E07 (L-Histidine)	6622.7500
822	Ax6 - Ax3	4.253347e-05	E07 (L-Histidine)	9877.0750
842	Ax3 - Ax1	8.813534e-03	E09 (L-Serine)	2909.2378
884	Ax6 - Ax4	1.284992e-02	E11 (Guanidine Hydrochloride)	-2728.7542
887	Ax3 - Ax1	4.627627e-03	E12 (Niaproof)	-6119.8767
891	Ax3 - Ax2	3.586907e-02	E12 (Niaproof)	-5507.0313
895	Ax4 - Ax3	1.687011e-03	E12 (Niaproof)	7418.2396
896	Ax5 - Ax3	1.537923e-02	E12 (Niaproof)	6894.6979
897	Ax6 - Ax3	1.876146e-02	E12 (Niaproof)	6180.1438
914	Ax6 - Ax4	2.856123e-02	F01 (Pectin)	-2269.4354
929	Ax6 - Ax4	9.983826e-04	F02 (D-Galacturonic Acid)	-1747.8458
935	Ax6 - Ax1	2.527683e-02	F03 (L-Galactonic Acid-g-Lactone)	-1440.3767
939	Ax6 - Ax2	1.374244e-02	F03 (L-Galactonic Acid-g-Lactone)	-1881.0179
944	Ax6 - Ax4	3.442240e-04	F03 (L-Galactonic Acid-g-Lactone)	-2595.3646
947	Ax3 - Ax1	1.981948e-03	F04 (D-Gluconic Acid)	4730.7517
949	Ax5 - Ax1	4.275801e-02	F04 (D-Gluconic Acid)	2922.0226
951	Ax3 - Ax2	2.674659e-02	F04 (D-Gluconic Acid)	4125.6161
955	Ax4 - Ax3	1.508796e-02	F04 (D-Gluconic Acid)	-4471.1979
965	Ax6 - Ax1	4.881676e-02	F05 (D-Glucuronic Acid)	-893.7913
974	Ax6 - Ax4	2.107920e-03	F05 (D-Glucuronic Acid)	-1534.2062
989	Ax6 - Ax4	1.122511e-03	F06 (Glucuronamide)	-2133.6625
995	Ax6 - Ax1	1.962862e-04	F07 (Mucic Acid)	-4822.2597
999	Ax6 - Ax2	2.762763e-04	F07 (Mucic Acid)	-5795.0821
1004	Ax6 - Ax4	6.793946e-04	F07 (Mucic Acid)	-5679.7771
1022	Ax3 - Ax1	9.740764e-03	F09 (D-Saccharic Acid)	3486.3628
1025	Ax6 - Ax1	0.000000e+00	F09 (D-Saccharic Acid)	-9493.9622
1029	Ax6 - Ax2	0.000000e+00	F09 (D-Saccharic Acid)	-9951.8429
1032	Ax6 - Ax3	0.000000e+00	F09 (D-Saccharic Acid)	-12980.3250
1034	Ax6 - Ax4	0.000000e+00	F09 (D-Saccharic Acid)	-11042.8563
1035	Ax6 - Ax5	0.000000e+00	F09 (D-Saccharic Acid)	-11022.2833
1064	Ax6 - Ax4	2.279043e-02	F11 (Tetrazolium Violet)	-1097.5167

```
1119  Ax6 - Ax2 4.127134e-02  G03 (D-Lactic Acid Methyl Ester)                   -789.6839
1124  Ax6 - Ax4 1.019538e-02  G03 (D-Lactic Acid Methyl Ester)                   -949.4458
1130  Ax6 - Ax1 1.065427e-07             G04 (L-Lactic Acid)                    -4733.1608
1133  Ax5 - Ax2 7.517423e-03             G04 (L-Lactic Acid)                     3955.5565
1134  Ax6 - Ax2 3.816310e-03             G04 (L-Lactic Acid)                    -3553.6268
1137  Ax6 - Ax3 1.988135e-05             G04 (L-Lactic Acid)                    -6962.9750
1139  Ax6 - Ax4 5.492458e-06             G04 (L-Lactic Acid)                    -5264.3396
1140  Ax6 - Ax5 1.480861e-06             G04 (L-Lactic Acid)                    -7509.1833
1175  Ax6 - Ax1 0.000000e+00              G07 (D-Malic Acid)                   -10842.6969
1179  Ax6 - Ax2 0.000000e+00              G07 (D-Malic Acid)                    -9991.7750
1182  Ax6 - Ax3 0.000000e+00              G07 (D-Malic Acid)                   -12097.5875
1184  Ax6 - Ax4 0.000000e+00              G07 (D-Malic Acid)                   -11520.9833
1185  Ax6 - Ax5 0.000000e+00              G07 (D-Malic Acid)                   -11091.2750
1205  Ax6 - Ax1 4.634296e-02       G09 (Bromo-Succinic Acid)                     1801.5490
1209  Ax6 - Ax2 3.065268e-02       G09 (Bromo-Succinic Acid)                     2325.3161
1217  Ax3 - Ax1 1.142895e-02            G10 (Nalidixic Acid)                    -4296.2986
1218  Ax4 - Ax1 4.016228e-02            G10 (Nalidixic Acid)                    -2273.7569
1220  Ax6 - Ax1 1.143290e-06            G10 (Nalidixic Acid)                    -4728.4424
1224  Ax6 - Ax2 7.303560e-03            G10 (Nalidixic Acid)                    -3605.4250
1231  Ax2 - Ax1 9.934547e-03          G11 (Lithium Chloride)                    -3788.0139
1234  Ax5 - Ax1 3.261184e-05          G11 (Lithium Chloride)                    -7942.3264
1236  Ax3 - Ax2 9.620386e-03          G11 (Lithium Chloride)                     7373.9063
1239  Ax6 - Ax2 4.291073e-02          G11 (Lithium Chloride)                     4586.7250
1241  Ax5 - Ax3 1.441401e-04          G11 (Lithium Chloride)                   -11528.2187
1243  Ax5 - Ax4 1.736738e-02          G11 (Lithium Chloride)                    -6141.7917
1245  Ax6 - Ax5 2.360336e-04          G11 (Lithium Chloride)                     8741.0375
1250  Ax6 - Ax1 2.990879e-03       G12 (Potassium Tellurite)                    -5091.0712
1263  Ax4 - Ax1 3.822043e-02                  H01 (Tween 40)                      876.8073
1277  Ax3 - Ax1 1.087496e-10     H02 (g-Amino-n-Butyric Acid)                    7491.7031
1280  Ax6 - Ax1 2.862465e-10     H02 (g-Amino-n-Butyric Acid)                    4744.3656
1281  Ax3 - Ax2 6.558964e-10     H02 (g-Amino-n-Butyric Acid)                    7774.0268
1284  Ax6 - Ax2 5.182124e-08     H02 (g-Amino-n-Butyric Acid)                    5026.6893
1285  Ax4 - Ax3 2.609634e-08     H02 (g-Amino-n-Butyric Acid)                   -7515.0313
1286  Ax5 - Ax3 3.942225e-06     H02 (g-Amino-n-Butyric Acid)                   -6760.0625
1289  Ax6 - Ax4 1.975029e-06     H02 (g-Amino-n-Butyric Acid)                    4767.6937
1290  Ax6 - Ax5 1.424554e-03     H02 (g-Amino-n-Butyric Acid)                    4012.7250
1309  Ax5 - Ax1 2.495324e-02     H04 (b-Hydroxy-Butyric Acid)                    2837.5087
1310  Ax6 - Ax1 2.557003e-02     H04 (b-Hydroxy-Butyric Acid)                    2248.3670
1313  Ax5 - Ax2 2.627254e-02     H04 (b-Hydroxy-Butyric Acid)                    3240.7560
1314  Ax6 - Ax2 3.593978e-02     H04 (b-Hydroxy-Butyric Acid)                    2651.6143
1400  Ax6 - Ax1 1.254185e-02                 H10 (Aztreonam)                    -2626.1382
1404  Ax6 - Ax2 1.962961e-03                 H10 (Aztreonam)                    -3765.1089
1409  Ax6 - Ax4 6.078472e-04                 H10 (Aztreonam)                    -4221.5583
1410  Ax6 - Ax5 3.860701e-03                 H10 (Aztreonam)                    -4465.9958
```

4.1. Two exemplary wells

In order give a better insight into the summary of the **multcomp** statistics as shown above, here, the pairwise comparison of all groups Ax1 to Ax6 will be demonstrated in detail below for two exemplary wells.

The code below plots the curve kinetics of two exemplary wells, D09 and F09 (Figure 3).

```
R> xy_plot(achromobacter[,,c("D09", "F09")],col = c("white", "grey", "blue", "black",
                           "green", "orange", "red"),
          include = list("MLSTcluster"), lwd = 0.4,
          neg.ctrl = NULL, legend.fmt  = list(space = "right"))
```

Apply **multcomp** statistics (Hothorn *et al.* 2008; Herberich *et al.* 2010) to the AUC curve parameter values from well ***D09***. The stars indicate the level of statistical significance between pairwise comparison of groups.

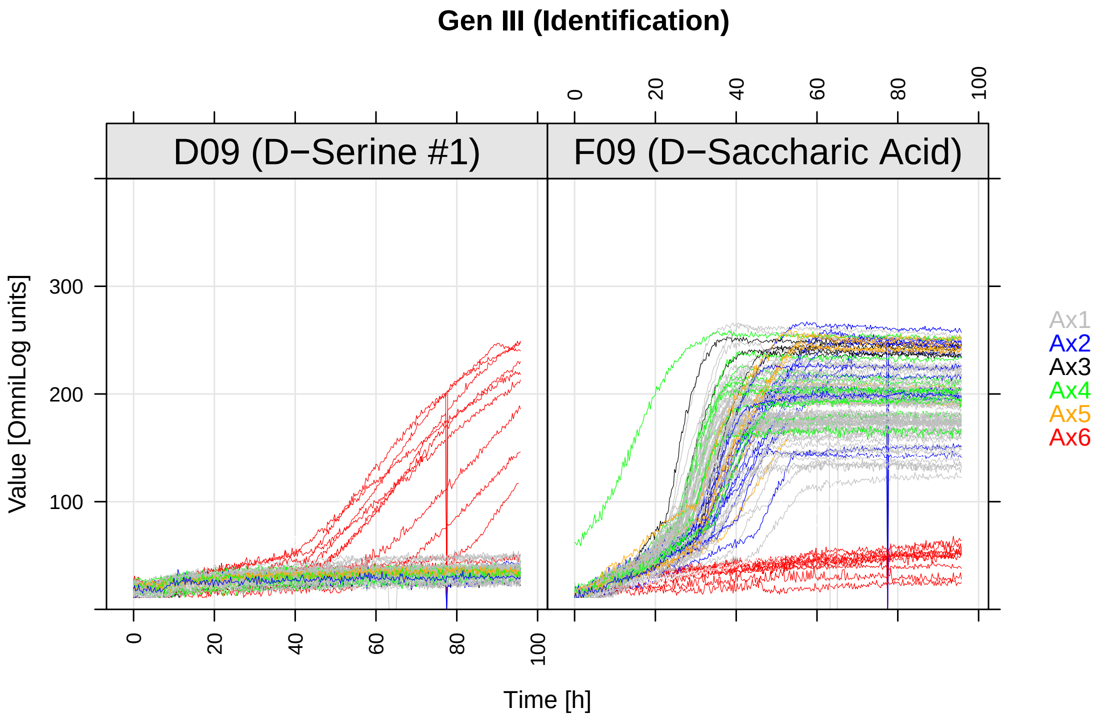

Figure 3: Curve kinetics of two selected wells.

```
R> achr1 <- subset(achr, query = list(MLSTcluster = "Ax_none"), invert = TRUE)
R> specificWellD09 <- opm_mcp(achr1[,,"D09"], model = ~ MLSTcluster,
                              m.type = "aov", linfct = c(Tukey = 1), subset = "AUC")
R> mcp.summary <- summary(specificWellD09)
R> mcp.summary$model$call <- NULL # avoid some unnecessary output
R> mcp.summary


         Simultaneous Tests for General Linear Hypotheses

Multiple Comparisons of Means: Tukey Contrasts


Linear Hypotheses:
               Estimate Std. Error t value Pr(>|t|)
Ax2 - Ax1 == 0  -86.682    237.083  -0.366    0.999
Ax3 - Ax1 == 0 -202.646    416.957  -0.486    0.996
Ax4 - Ax1 == 0  146.427    253.083   0.579    0.991
Ax5 - Ax1 == 0    1.146    344.894   0.003    1.000
Ax6 - Ax1 == 0 4292.067    273.919  15.669   <1e-05 ***
Ax3 - Ax2 == 0 -115.964    460.175  -0.252    1.000
Ax4 - Ax2 == 0  233.109    319.310   0.730    0.975
Ax5 - Ax2 == 0   87.827    396.056   0.222    1.000
Ax6 - Ax2 == 0 4378.748    336.064  13.029   <1e-05 ***
Ax4 - Ax3 == 0  349.073    468.619   0.745    0.973
Ax5 - Ax3 == 0  203.792    523.932   0.389    0.999
Ax6 - Ax3 == 0 4494.712    480.192   9.360   <1e-05 ***
Ax5 - Ax4 == 0 -145.281    405.836  -0.358    0.999
Ax6 - Ax4 == 0 4145.640    347.537  11.929   <1e-05 ***
Ax6 - Ax5 == 0 4290.921    419.146  10.237   <1e-05 ***
---
Signif. codes:  0 '***' 0.001 '**' 0.01 '*' 0.05 '.' 0.1 ' ' 1
(Adjusted p values reported -- single-step method)
```

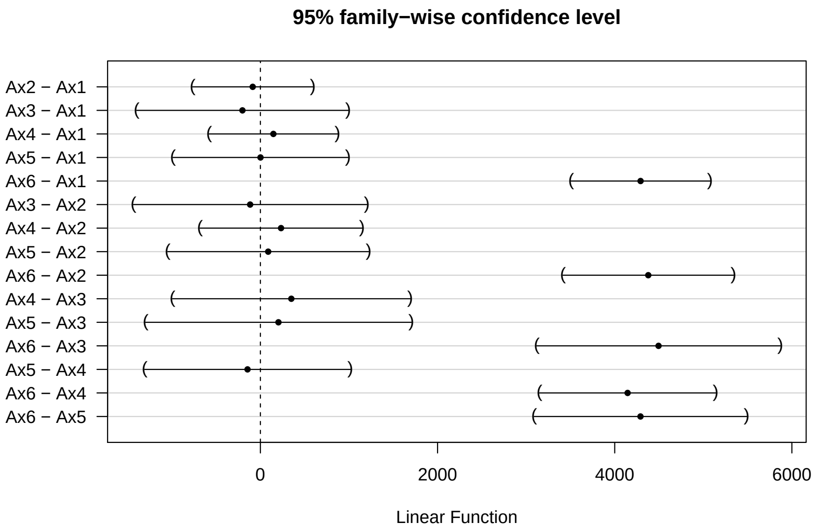

Figure 4: Graphical depiction of the results from the multiple comparison for well D09. On the y axis the performed comparisons are indicated as differences of the groups, determining which differences of means are computed. All pairwise comparisons are shown. The filled black circle indicates the point estimator of difference between the mean of groups. 95% confidence intervals are indicated by horizontal bars and parentheses. In pairwise comparisons, if the 95% confidence interval includes zero (dashed vertical line) there is no significant difference between the group means. Conversely, if zero is not included, a significant difference is indicated. Furthermore, the more distant the 95% confidence interval is from zero, the larger the biological effect size, i.e. the real difference between the groups.

The code below plots the results from the multiple comparison for Well D09 (Figure 4).

```
R> plot(specificWellD09)
```

Apply **multcomp** statistics (Hothorn *et al.* 2008; Herberich *et al.* 2010) to the AUC curve parameter values from well ***F09***. The stars indicate the level of statistical significance between pairwise comparison of groups.

```
R> specificWellF09 <- opm_mcp(achr1[,,"F09"], model = ~ MLSTcluster,
                            m.type = "aov", linfct = c(Tukey = 1), subset = "AUC")
R> mcp.summary <- summary(specificWellF09)
R> mcp.summary$model$call <- NULL # avoid some unnecessary output
R> mcp.summary
```

```
         Simultaneous Tests for General Linear Hypotheses

Multiple Comparisons of Means: Tukey Contrasts


Linear Hypotheses:
                Estimate Std. Error t value Pr(>|t|)
Ax2 - Ax1 == 0    457.88     579.10   0.791  0.96467
Ax3 - Ax1 == 0   3486.36    1018.46   3.423  0.00984 **
Ax4 - Ax1 == 0   1548.89     618.18   2.506  0.12031
Ax5 - Ax1 == 0   1528.32     842.44   1.814  0.43690
Ax6 - Ax1 == 0  -9493.96     669.07 -14.190  < 0.001 ***
```

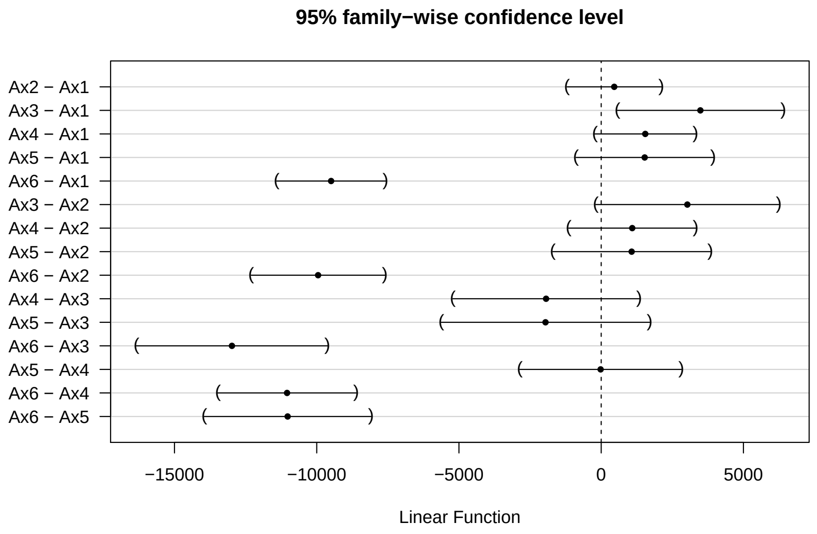

Figure 5: Graphical depiction of the results from the multiple comparison for well F09.

```
Ax3 - Ax2 == 0   3028.48    1124.02   2.694  0.07674 .
Ax4 - Ax2 == 0   1091.01     779.94   1.399  0.70710
Ax5 - Ax2 == 0   1070.44     967.40   1.107  0.86559
Ax6 - Ax2 == 0  -9951.84     820.87 -12.124  < 0.001 ***
Ax4 - Ax3 == 0  -1937.47    1144.65  -1.693  0.51535
Ax5 - Ax3 == 0  -1958.04    1279.75  -1.530  0.62277
Ax6 - Ax3 == 0 -12980.32    1172.91 -11.067  < 0.001 ***
Ax5 - Ax4 == 0    -20.57     991.29  -0.021  1.00000
Ax6 - Ax4 == 0 -11042.86     848.89 -13.009  < 0.001 ***
Ax6 - Ax5 == 0 -11022.28    1023.80 -10.766  < 0.001 ***
---
Signif. codes:  0 '***' 0.001 '**' 0.01 '*' 0.05 '.' 0.1 ' ' 1
(Adjusted p values reported -- single-step method)
```

The code below plots the results from the multiple comparison for Well F09 (Figure 5).

```
R> plot(specificWellF09)
```

References

Bochner B (2009). "Global Phenotypic Characterization of Bacteria." *FEMS Microbiological Reviews*, **33**, 191–205.

Bochner B, Gadzinski P, Panomitros E (2001). "Phenotype MicroArrays for High Throughput Phenotypic Testing and Assay of Gene Function." *Genome Research*, **11**, 1246–1255.

Borcard D, Legendre P, Gillet F (2011). *Numerical Ecology with R*. Springer, New York. ISBN 9781441979759 1441979751. URL http://www.worldcat.org/search?qt=worldcat_org_all&q=9781441979759.

Herberich E, Sikorski J, Hothorn T (2010). "A Robust Procedure for Comparing Multiple Means under Heteroscedasticity in Unbalanced Designs." *PLoS ONE*, **5**(3), e9788.

Hothorn T, Bretz F, Westfall P (2008). "Simultaneous Inference in General Parametric Models." *Biometrical Journal*, **50**, 346–363. See vignette("generalsiminf", package = "multcomp").

Legendre P, Legendre L (1998). *Numerical Ecology*. Developments in Environmental Modelling. Elsevier Science. ISBN 9780080537870. URL http://books.google.de/books?id=KBoHuoNRO5MC.

Leisch F (2002). "Sweave: Dynamic Generation of Statistical Reports Using Literate Data Analysis." In W Härdle, B Rönz (eds.), *Compstat 2002 — Proceedings in Computational Statistics*, pp. 575–580. Physica Verlag, Heidelberg. ISBN 3-7908-1517-9, URL http://www.stat.uni-muenchen.de/~leisch/Sweave.

Vaas L, Sikorski J, Michael V, Göker M, Klenk H (2012). "Visualization and Curve Parameter Estimation Strategies for Efficient Exploration of Phenotype MicroArray Kinetics." *PLoS ONE*, **7**, e34846. See vignette("opm.pdf", package = "opm"), URL http://cran.r-project.org/web/packages/opm/vignettes/opm.pdf.

Vaas LAI, Sikorski J, Hofner B, Fiebig A, Buddruhs N, Klenk HP, Göker M (2013). "opm: an R package for analysing OmniLog phenotype microarray data." *Bioinformatics*, **29**(14), 1823–1824. doi:10.1093/bioinformatics/btt291. http://bioinformatics.oxfordjournals.org/content/29/14/1823.full.pdf+html, URL http://bioinformatics.oxfordjournals.org/content/29/14/1823.abstract.

Affiliation:

Johannes Sikorski
Leibniz Institute DSMZ – German Collection of Microorganisms and Cell Cultures
Braunschweig

URL: www.dsmz.de
